# New Sesterterpenes from the Antarctic Sponge *Suberites* sp.

**DOI:** 10.3390/md22120551

**Published:** 2024-12-06

**Authors:** Stine S. H. Olsen, Sydney K. Morrow, Julia L. Szabo, Michael N. Teng, Kim C. Tran, Charles D. Amsler, James B. McClintock, Bill. J. Baker

**Affiliations:** 1 Department of Chemistry, University of South Florida, 4202 E. Fowler Avenue, CHE205, Tampa, FL 33620, USA; olsens@usf.edu (S.S.H.O.); kalyn12@usf.edu (S.K.M.); julia139@usf.edu (J.L.S.); 2 Department of Internal Medicine, University of South Florida, Tampa, FL 33612, USA; mteng@usf.edu (M.N.T.); kteng@usf.edu (K.C.T.); 3 Department of Biology, University of Alabama at Birmingham, 1300 University Blvd, Birmingham, AL 35233, USA; amsler@uab.edu (C.D.A.); mcclinto@uab.edu (J.B.M.)

**Keywords:** *Suberites* sp., sesterterpenes, suberitane, porifera, Antarctica, RSV

## Abstract

Chemical investigation of the Antarctic sponge *Suberites* sp. has previously led to the identification of new suberitane derivatives, some of which show bioactivity toward respiratory syncytial virus (RSV). Our ongoing NMR-guided investigation of new specimens of the sponge resulted in the isolation of five new analogs (**1**–**5**), previously reported suberitenones A–D (**6**–**9**), and oxaspirosuberitenone (**10**). Suberitenone K (**1**) was characterized as the 8-keto derivative of **6**, while three new phenols, suberitandiol (**2**), abeosuberitandiol (**3**), and furanosuberitandiol (**4**), and the degraded sesterterpene norsuberitenone B (**5**) were also found. Compound **3** displays a ring contraction while **4** has a new dihydrofuran ring. Structural characterization was achieved by a combination of NMR, HR-MS, and X-ray diffraction (XRD). Moderate activity towards RSV was reported for **9** and the new metabolite **1**, with IC_50_ values of 15.0 μM and 39.8 μM, respectively.

## 1. Introduction

With an expansive range and biogeographic isolation, the waters of the Southern Ocean surrounding Antarctica host rich and diverse marine benthic invertebrate communities [1,2]. Marine sponges often act as the dominant taxa in many benthic habitats in the region, and these organisms often produce chemical defenses to survive in communities structured by biotic influences like predation and competition for limited resources [2]. Sponges from Antarctica have been found to produce a wide variety of chemical compounds, including terpenes [3]. Naturally occurring terpenes have interesting chemical structures that exhibit antimicrobial, anticancer, and anti-inflammatory properties; as such, they may have pharmaceutical applications in the field of drug discovery [4,5,6]. The isolation of these compounds reveals auspicious results for bioprospecting efforts in Antarctica [4].

*Suberites* is a sponge genus of the family Suberitidae. These organisms can be found across various oceanic habitats, including coral reefs, rocky intertidal zones, and deep sea environments [2]. *Suberites* spp. play an important role in the ecosystems they inhabit through their water filtration and benthic shaping qualities [2]. The genus *Suberites* belongs to the largest and most diverse class of sponges, Demospongiae, which leaves room for vast research to be conducted on the natural product compounds they possess. Our research group has previously studied Antarctic *Suberites* sp. and discovered a variety of unreported sesterterpenes, including neosuberitenone, which was characterized by a new carbon scaffold, as well as six suberitenone derivatives, an ansellane-type terpenoid, and a highly degraded sesterterpene [7]. Some of these compounds were reported with antiviral activity [7].

Herein, we report on our continuing investigation of the Antarctic sponge *Suberites* sp. New specimens of the sponge were subject to ^1^H NMR-guided purification using reverse-phase high-performance liquid chromatography (HPLC), resulting in the isolation of five new suberitane derivatives, three being derivatives of suberiphenol [8]. Additional supplies of our previously published suberitenones were isolated together with two major metabolites (suberitenone A (**6**) and B (**7**)), minor metabolites (suberitenone C (**8**) and D (**9**)), and oxaspirosuberitenone (**10**) [7,8,9,10]. We report the structure elucidation of the previously unreported compounds and their RSV antiviral activity.

## 2. Results and Discussion

**Figure 1:**
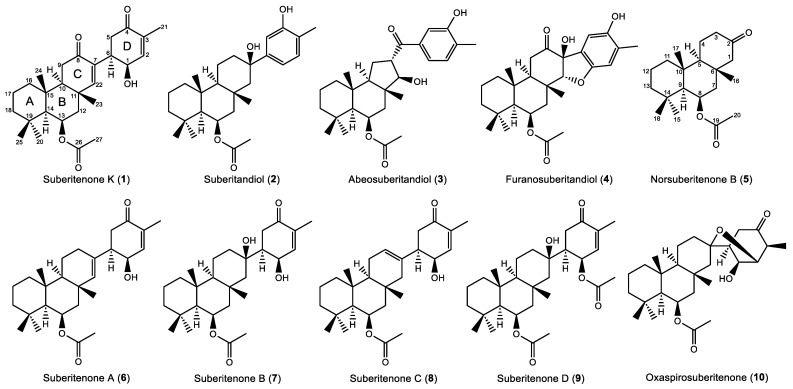
Unreported (**1**–**5**) and known (**6**–**10**) sesterterpenes isolated from the Antarctic sponge *Suberites* sp.

The molecular formula of suberitenone K (**1**) was determined to be C_27_H_38_O_5_ based on HRESIMS data ([M + Na]^+^ *m*/*z* 465.2622), corroborated by the ^1^H and ^13^C NMR spectra (Table 1). The ^13^C NMR spectrum indicated **1** to have four olefinic carbons, including two methines and two olefinic quaternary signals, as well as three carbonyls. These account for five degrees of unsaturation, indicating that **1** has a four-ring system. The remaining twenty carbons are accounted for by three quaternary carbons, three aliphatic methines, two deshielded methines, six methyl groups, and six methylene groups. The ^1^H NMR data (Table 1) showed two oxygen-bearing methines at δ_H_ 4.25 (H-1) and δ_H_ 5.56 (H-13), two olefinic methines at δ_H_ 6.80 (H-2) and δ_H_ 6.40 (H-22), three aliphatic methines at δ_H_ 3.35 (H-6), δ_H_ 1.96 (H-10), and δ_H_ 1.23 (H-14), and six singlet methyl groups. These NMR data indicate this compound to be structurally related to other reported compounds from *Suberites* sp. (Figure 1), known as the suberitane carbon skeleton [9].

**Figure 2:**
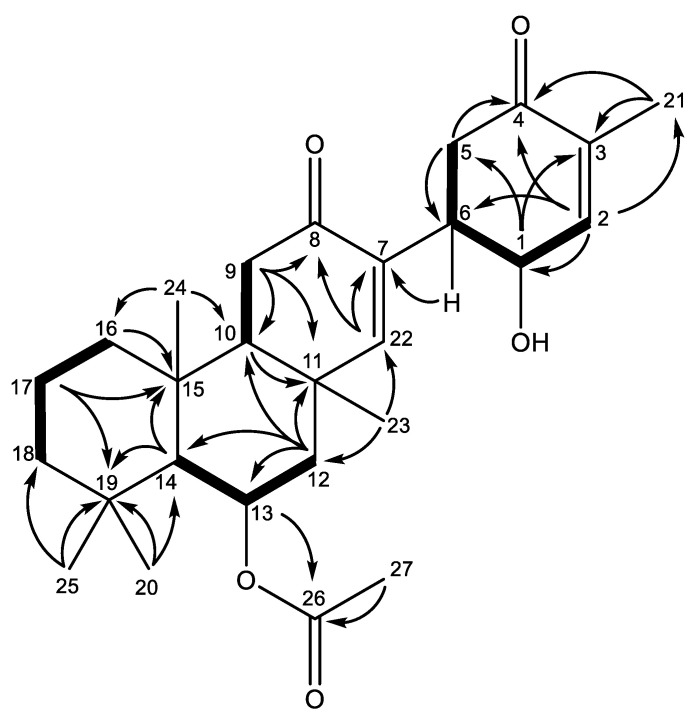
Key COSY (bold) and HMBC (arrow) correlations of **1**.

Ring A’s partial structure was established by a combination of COSY and HMBC correlations (Figure 1 and Figure 2). The geminal methyl groups H_3_-20 and H_3_-25 (δ_H_ 0.94, 1.06) anchor the spin system, both displaying HMBC correlations to C-14, C-18, and C-19 (δ_C_ 57.3, 45.3, and 35.1, respectively). The methine H-14 (δ_H_ 1.23) displayed HMBC correlations to methyl groups C-20 (δ_C_ 33.3) and C-24 (δ_C_ 17.4) and the quaternary carbons C-15 (δ_C_ 38.2) and C-19. H_3_-24 (δ_H_ 1.34) had HMBC correlations to C-10 (δ_C_ 54.9), C-14, C-15, and methylene C-16 (δ_C_ 42.2). The COSY data further connect H_2_-17 (δ_H_ 1.81/1.51) to H_2_-16 (δ_H_ 1.69/0.98) and H_2_-18 (δ_H_ 1.41/1.26), resulting in ring A.

Extending the spin system, COSY correlations were observed between H-14 and oxymethine H-13 (δ_H_ 5.59), which further correlated with H_2_-12a/12b (δ_H_ 2.06/1.66). H_2_-12 had HMBC correlations to C-10, C-11 (δ_C_ 37.7), C-13 (δ_C_ 72.0), and C-14, closing the six-membered ring B with quaternary carbon C-15 between C-10 and C-14 based on the previously described HMBC correlation between H_3_-24 and C-10. The singlet methyl H_3_-23 (δ_H_ 1.29) had HMBC correlations to C-10, C-11, C-12, and the olefinic methine C-22 (δ_C_ 160.7), placing the methyl on quaternary carbon C-11. H_2_-9 (δ_H_ 2.52/2.50) showed HMBC correlations to carbonyl C-8 (δ_C_ 202.1), C-10, and C-11. HMBC correlations from H-22 to C-8 and the olefinic quaternary carbon C-7 (δ_C_ 134.2) connect carbonyl C-8 and olefinic methine C-22 with C-7, completing ring C.

A separate spin system comprising ring D was constructed based on the COSY correlations from oxymethine H-1 (δ_H_ 4.25) to aliphatic methine H-6 (δ_H_ 3.35) and olefinic methine H-2 (δ_H_ 6.80). Further, H-2 showed HMBC correlations to C-1 (δ_C_ 65.2), C-4 (δ_C_ 202.2), C-6 (δ_C_ 38.5), and C-21 (δ_C_ 15.7). The singlet methyl group H_3_-21 (δ_H_ 15.7) displayed HMBC correlations to C-2 (δ_C_ 145.3), C-3 (δ_C_ 137.2), and C-4, placing it on the quaternary carbon C-3 with olefinic methine (C-2) and ketone (C-4) on each side. The non-equivalent methylene H_2_-5a/5b (δ_H_ 2.75/2.15) had HMBC correlations with C-1, C-4, and C-6, closing ring D. This was supported by COSY correlations from methine H-6 to H-1 and H_2_-5a/5b. 

The two spin systems were joined based on the HMBC correlation between H-5a and H-6 with olefinic quaternary carbon C-7 (δ_C_ 134.2), establishing the suberitane backbone. The acetoxy group can be placed on oxygen-bearing C-13 based on the deshielded shift of both its carbon (δ_C_ 72.0) and proton (δ_H_ 5.59) and HMBC correlations from H-13 to C-26 (δ_C_ 172.1), completing the planar structure of **1**. 

The relative stereochemistry was determined by key NOE correlations between H_3_-20, H-13, and H-14, and further between H-14 and H-10. H_3_-23 has an NOE correlation to the acetoxy methyl, placing these on the same face of the ring. H_3_-25 has an NOE correlation to H_3_-24, placing these on the same face as the acetoxy, as H_3_-25 is opposite H_3_-20. The relationships of H-1 and H-6 were determined to be *syn* to each other based on their coupling constants of *J* = 4.0. NOE correlations, coupling constants, and a negative optical rotation were compared to the published data for suberitenone A (**6**) and used to establish **1** as sharing stereocenters in the same orientation, suggesting the same absolute configuration of 1*R*,6*R*,10*S*,11*S*,13*R*,14*S*,15*R*, as previously determined by Shin et al. [9] for **6** using a modified Mosher’s method and CD analysis, as well as our analysis of suberitenone E, which was confirmed by XRD [7].

**Figure 3:**
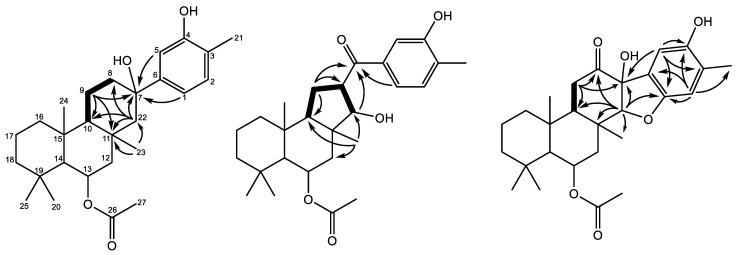
Key COSY (bold) and HMBC (arrows) correlations of **2**, **3**, and **4**, respectively, highlighting changes in rings C and D.

Suberitandiol (**2**) was determined to have the molecular formula C_27_H_40_O_4_ by the negative ion formate adduct ([M + CHOO]^−^ 473.2908 *m*/*z*). The NMR data of **2** (Table 2) suggest rings A and B to be as found in **1**, with changes in rings C and D as highlighted in Figure 3. The changes in ring C are supported by HMBC correlations from H_3_-23 (δ_H_ 1.43) methyl to C-10 (δ_C_ 58.9), C-11 (δ_C_ 34.8), C-12 (δ_C_ 46.7), and C-22 (δ_C_ 57.2) being placed on the quaternary carbon C-11 and connecting ring B and C, as shown for **1**. C-22 for **1** was an olefinic methine, but for **2** a non-equivalent methylene was found. Ring C was further verified and connected through H-22a (δ_H_ 1.57) with HMBC correlations to C-8 (δ_C_ 41.1), C-10, C-11, C-23 (δ_C_ 22.7), and quaternary carbon C-7 (δ_C_ 74.4), as shown in Figure 3. Methylene H-9a (δ_H_ 1.86) displays COSY correlations to H_2_-8 (δ_H_ 1.90) and H-10 (δ_H_ 1.06), establishing much of ring C. Ring C was completed between C-8 and C-9 by H-9b (δ_H_ 1.69), which demonstrated HMBC correlations to C-7, C-10, and C-11. With ring D left, three olefinic/aromatic methines, one deshielded methyl group, three quaternary carbons, and two oxygens remain left to account. The three olefinic/aromatic methines and three quaternary carbons make up an aromatic ring with three substituents. Methine H-2 (δ_H_ 7.07 (d)) had COSY correlations to methine H-1 (δ_H_ 6.90), creating an olefinic bond and suggesting the loss of the oxygen-bearing methine previously observed in **1**. Further, H-2 had HMBC correlations to the quaternary carbons C-4 (δ_C_ 152.6) and C-6 (δ_C_ 149.5) and the methyl C-21 (δ_C_ 15.3), as observed for **1.** The third methine (H-5, δ_H_ 6.95) is a singlet with HMBC correlations to C-1 (δ_C_ 116.6) and C-3 (δ_C_ 122.0). The H_3_-21 methyl (δ_H_ 2.23) had HMBC correlations to C-2 (δ_C_ 130.7), C-3 (δ_C_ 122.0), and C-4, placing the methyl on the quaternary carbon C-3. The aromatic methines H-1 and H-5 both have HMBC correlations to C-7, suggesting a bridge between C-6 and C-7, connecting ring C and D. This leaves an open valence on C-4 and C-7, with two oxygens and two protons left to account for, indicating a hydroxyl group on each to complete the planar structure of **2**. These assignments are supported by their deshielded shifts. The 3D structure was solved by comparing the NOE and coupling constants to **1**. The tertiary alcohol at C-7 was deduced by NOE correlations in DMSO-*d*_6_ from OH-7 (δ_H_ 4.38) to H_3_-23 (δ_H_ 1.34). The absolute configuration is proposed as 7*R*,10*S*,11*S*,13*R*,14*S*,15*R* based on similarity to the known compound suberitenone B (**7**) [9]. 

Abeosuberitandiol (**3**) was determined to have the molecular formula C_27_H_38_O_5_, as established by its ^1^H and ^13^C NMR data (Table 2) and the detection of its formate anion adduct at 487.2697 *m*/*z*. The three aromatic methines and its 2D data (Figures S27–S29) indicate **3** to have rings A, B, and D, as found for **2**. Figure 3 illustrates the HMBC and COSY correlations, highlighting the structural differences observed in ring C. H_3_-23 (δ_H_ 1.16) has HMBC correlations to C-10 (δ_C_ 56.4), C-12 (δ_C_ 42.6), and C-22 (δ_C_ 83.5), as shown for the other structures; however, differently from **1** and **2**, H-22 (δ_H_ 4.02) has the shift of an oxygen-bearing methine. H-22 has a COSY correlation to methine H-7 (δ_H_ 3.58), which further shows COSY correlations to the non-equivalent methylene H_2_-9a/9b (δ_H_ 2.09/1.66). H_2_-9a/9b has a COSY correlation to H-10 (δ_H_ 1.30), completing ring C as a five-membered ring. H-9a has HMBC correlations to C-7 (δ_C_ 50.1), C-10 (δ_C_ 56.4), and the carbonyl C-8 (δ_C_ 202.1). H-22 and H-1 (δ_H_ 7.43) display HMBC correlations to C-8, as shown in Figure 3, suggesting that ketone acts as a bridge between ring C and D. The absolute configuration was deduced previously, showing the relative orientation of chiral centers to be 10R*,11R*,13R*,14S*,15R*. The NOE correlations between H-7/H_3_-23 and H-10/H-22 place H-7 and H-22 anti to one another, suggesting the absolute configuration of **3** to be 7*S*,10*R*,11*R*,13*R*,14*S*,15*R*,22*R*.

Furanosuberitandiol (**4**) was found with the molecular formula C_27_H_36_O_6_, determined by the ^1^H and ^13^C NMR data (Table 2) and (−)HRESIMS ([M + CHOO]^−^ 501.2489 *m*/*z*). The formula suggests the structure has ten degrees of unsaturation. The NMR data indicate **4** to have an aromatic ring, as observed for **2** and **3**, and two carbonyl groups. This leaves **4** with five rings. Figure 3 highlights the key HMBC and COSY correlations to establish the aromatic ring, and the further differences in ring C. Its NMR data (Table S4) suggest **4** to have the same A/B ring system observed for **1**. Ring D differs in having two aromatic methines: C-2 (δ_C_ 112.1) and C-5 (δ_C_ 109.7). For **2** and **3**, C-1 was observed as a methine; however, it is found in **4** as a quaternary carbon with a deshielded shift (δ_C_ 155.3), suggesting oxygen substitution. H_3_-23 (δ_H_ 1.00) had HMBC correlations to C-10 (δ_C_ 45.9) and C-22 (δ_C_ 97.4), suggesting C-22 to be oxygen-bearing, as shown in **3**. H-22 (δ_H_ 4.19) displays HMBC correlations to C-1 (δ_C_ 155.3), C-7 (δ_C_ 83.3), C-8 (δ_C_ 210.8), C-10 (δ_C_ 45.9), C-11 (δ_C_ 38.3), and C-23 (δ_C_ 18.9), suggesting a bridge between C-1 and C-22 through oxygen. The non-equivalent methylene H-9a (δ_H_ 2.48) had a COSY correlation to H-10 (δ_H_ 1.88) and HMBC correlations to quaternary carbon C-7 (δ_C_ 83.3) and carbonyl C-8 (δ_C_ 210.8), establishing the further connectivity of ring C. The H-9a and H-22 correlations to C-7 and C-8 suggest the completion of ring C between C-7 and C-22. To complete the planar structure, a hydroxyl group is left to account for, and one and two valences are open on C-6 and C-7, respectively. This suggests the hydroxy group is placed on the oxygen-bearing C-7, creating a bridge between C-6 and C-7 and establishing the fifth ring system. NOE correlations and coupling constants, as described earlier, were used to propose the absolute configuration of **5**. The key NOE correlations between H_3_-23 and H-22 determined a *syn* relationship for these protons, while the C-7 tertiary alcohol was placed on the same face as H-22 and H_3_-23, as shown for **3** and **7** [9]. This was corroborated by key NOE correlations in (CD_3_)_2_SO between OH-7 and H-22 and a weak correlation between OH-7 and H_3_-23 (Figure S42), giving **5** the proposed absolute configuration of 7*S*,10*R*,11*R*,13*R*,14*S*,15*R*,22*R*.

**Figure 4:**
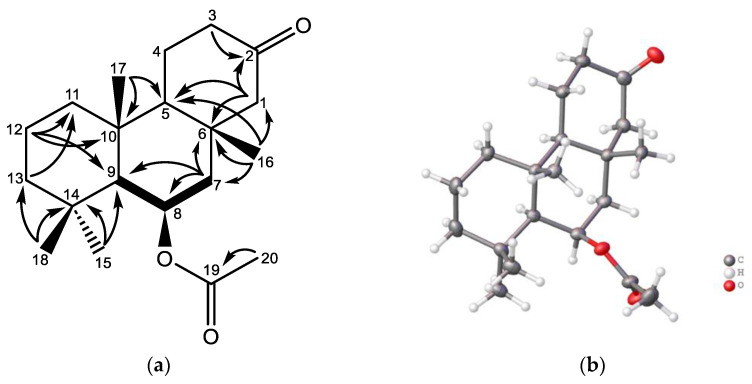
(**a**) Key COSY (bold) and HMBC (arrow) correlations of **5**; (**b**) XRD of **5**.

Norsuberitenone B (**5**) was isolated as a white crystal with the molecular formula C_20_H_32_O_3_ based on the *m*/*z* 321.2433 proton adduct established by (+)HRESIMS. Its formula indicates five degrees of unsaturation, with two being carbonyl groups, suggesting a Tthree-ring system. Its NMR data (Table S5) suggest **5** has a similar ring system to that shown for **2**, but lacking ring D. The methylenes H-1b (δ_H_ 2.03) and H-3b (δ_H_ 2.29) have HMBC correlations to a ketone (C-2: δ_C_ 210.9), suggesting a ketone to have replaced the tertiary alcohol bridging to ring D in **2**, establishing **5** to structurally relate to norsuberitenone A [7]. The stereochemistry was determined by key NOE correlations from H_3_-15 to H-8 (δ_H_ 5.52) and H-9 (δ_H_ 1.15), and further between H-9 and H-5 (δ_H_ 1.50). NOE correlations were observed between H_3_-16 and the acetyl methyl group. XRD (Figure 4) confirmed the structure and determined the absolute configuration to be 5*S*,6*S*,8*R*,9*S*,10*R*.

**Figure 5:**
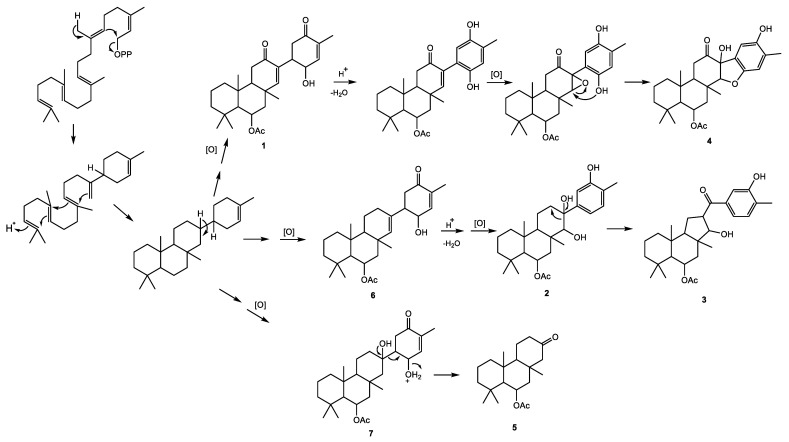
Proposed biogenesis of compounds **1**–**5**.

Biosynthetically, the suberitane class differs from many other sesterterpenes due to the pendant ring D [9]. The proposed biosynthetic pathway of the newly reported metabolites **1**–**5** is presented in Figure 5. The degraded suberitenone, **5**, could be rationalized as the elimination product of ring D from **7**. Compound **1** can be derived from the oxidation of a tetracyclic intermediate, as shown in Figure 5. The phenolic compounds **2**–**4** are perhaps derived from a protonation-catalyzed dehydration of the alcohol allylic to the α,β-unsaturated ketone.

Bracegirdle et al. reported RSV activity for the known compounds suberitenone A (**6**) and suberitenone B (**7**), with IC_50_ values of 7.8 μM and 3.2 μM, respectively, while the suberitenone compounds F, G, and H showed moderate activity [7]. Herein, we report moderate RSV activity for suberitenone D (**9**) (Table 3), while the others displayed no activity. A decrease in activity is shown with the degraded sesterterpene **5** relative to **7**, suggesting the presence of ring D to be important for further structure–activity relationship studies. Furthermore, no activity was shown for **2** and **3**, suggesting the importance of the α,β-unsaturated ketone on ring D. Suberitenone D (**9**) has a decrease in activity compared to **7**, with the only structural difference being the acetoxy replacing the hydroxyl group on ring D. Suberitenone C (**8**) showed no activity and **1** displayed moderate activity, suggesting that Δ^7(22)^ olefin is favorable over Δ^8^ olefin, but an α,β-unsaturated ketone on ring C will decrease the activity. Metabolite **9** showed an IC_50_ value of 35.8 μM, while none of the other metabolites displayed any cytotoxicity against A549 adenocarcinoma cells. All metabolites were screened for antibiotic activity against ESKAPE pathogens and antifungal activity against *Candida albicans* and *C. auris* and were found inactive. 

## 3. Materials and Methods

### 3.1. General Experimental Procedures

Optical rotations were measured using an AutoPol IV digital polarimeter at 589 nm with a 1 dm path length cell. UV/Vis spectra were extracted from HPLC chromatograms. NMR spectra were acquired using a Bruker Neo 400 MHz broadband spectrophotometer with a cryoprobe or a Bruker Neo 600 MHz broadband spectrophotometer. The residual solvent peaks were used as an internal chemical shift reference (CDCl_3_: δ_C_ 77.0; δ_H_ 7.27, MeOD: δ_C_ 49.0; δ_H_ 3.31, (CD_3_)_2_SO: δ_C_ 39.5; δ_H_ 2.50). High-resolution mass spectrometry–liquid chromatography data were obtained on an Agilent 6540 LC-MS QTOF coupled to an Agilent Jet-stream electrospray ionization detector. H_2_O (A) and 0.1% FA in CH_3_CN (B) were used as mobile phases in a Phenomenex Kinetex C18 column (2.6 μm, 100 Å, 150 × 3 mm: 0.5 mL/min). Reverse-phase HPLC was performed on a Shimadzu LC20-AT system equipped with a photodiode array detector (M20A) using a preparatory Phenomenex C18 column (5 μm, 100 Å, 250 × 21.2mm: 10 mL/min) or a semi-preparatory Phenomenex C18 column (10 μm, 100 Å, 250 × 10 mm: 4 mL/min). The methanol and acetonitrile used for column chromatography were obtained from Fisher Co. and were of HPLC grade (>99% purity). The H_2_O was distilled and filtered. Solvents mixtures are reported as *% v*/*v.*

### 3.2. Biological Materials, Extraction, and Isolation

Sponge specimens were collected from Palmer Station, Antarctica, in 2018. The frozen sponge (1.7 g) was freeze-dried before being extracted in MeOH twice overnight. An HP20 column was prepared by washing with (CH_3_)_2_CO and pre-equilibrated in H_2_O. The extract was passed through the column before the eluent was diluted with an equal volume of H_2_O and passed back through the column. The eluent was diluted a second time and passed through the column again. The column was eluted with 750 mL of (1) 30% Me_2_CO/H_2_O, (2) 75% Me_2_CO/H_2_O, and (3) 100% Me_2_CO. ^1^H NMR-guided fractionation was completed on the second fraction until pure compounds were achieved. Initial fractionation was performed using preparative C18 HPLC with a 70–100% ACN/H_2_O gradient over 15 min, resulting in 12 fractions. ^1^H NMR was performed on each fraction, prioritizing fractions with a methyl around 2 ppm and an oxygen-bearing methine around 4.5 ppm. Semipreparative C18 HPLC was performed on fraction 8 using a gradient of 50–100% MeOH/H_2_O, resulting in norsuberitenone B (**5**: 4.9 mg). The same method was used to purify **1**, **3**, and **6,** affording suberitenone K (**1**: 2.0 mg), furanosuberitandiol (**4**: 0.9 mg), and abeosuberitandiol (**3**: 1.2 mg), respectively. Fraction 11 was purified by semipreparative C18 HPLC using a gradient of 75–100% MeOH/H_2_O, resulting in suberitandiol (**2**: 3.2 mg) and the known compound suberitenone C (**8**). Fraction 10 was purified using the same method as described for fraction 11, resulting in the known compounds suberitenone D (**9**) and oxaspirosuberitenone (**10**). Fraction 9 and 12 contained the known compounds suberitenone B (**7**) and suberitenone A (**6**), respectively.

### 3.3. Spectroscopic Data for Suberites (**1**–**5**)

Suberitenone K (**1**): white film; ${\left[\alpha \right]}_{D}^{22}$ −31.1 (*c* 0.14, MeOH); UV (ACN/H_2_O) λ_max_ 229 nm; ^1^H NMR (600 MHz) and ^13^C NMR (150 MHz), Table S1; HRESIMS *m*/*z* 465.2616 [M + Na]^+^ (calcd. for C_27_H_38_O_5_Na, 465.2622; Δ 1.38 ppm).

Suberitandiol (**2**): white film; ${\left[\alpha \right]}_{D}^{22}$ −3.4 (*c* 0.32, MeOH); UV (ACN/H_2_O) λ_max_ 274 nm; ^1^H NMR (400 MHz) and ^13^C NMR (100 MHz), Table S2; HRESIMS *m*/*z* 473.2908 [M + CHOO]^−^ (calcd. for C_28_H_41_O_6_, 473.2908; Δ 0.13 ppm).

Abeosuberitandiol (**3**): white film; ${\left[\alpha \right]}_{D}^{22}$ +8.0 (*c* 0.09, MeOH); UV (ACN/H_2_O) λ_max_ 261, 310 (sh) nm; ^1^H NMR (400 MHz) and ^13^C NMR (100 MHz), Table S3; HRESIMS *m*/*z* 487.2697 [M + CHOO]^−^ (calcd. for C_28_H_38_O_7_, 487.2701; Δ 0.88 ppm).

Furanosuberitandiol (**4**): white film; ${\left[\alpha \right]}_{D}^{22}$ −8.6 (*c* 0.09, MeOH); UV (ACN/H_2_O) λ_max_ 261 nm; ^1^H NMR (400 MHz) and ^13^C NMR (100 MHz), Table S4; HRESIMS *m*/*z* 501.2489 [M + CHOO]^−^ (calcd. for C_28_H_37_O_8_, 501.2494; Δ 0.98 ppm).

Norsuberitenone B (**5**): white film ${\left[\alpha \right]}_{D}^{22}$+3.0 (*c* 0.24, MeOH); UV (ACN/H_2_O) λ_max_ 225 nm; ^1^H NMR (400 MHz) and ^13^C NMR (100 MHz), Table S5; HRESIMS *m*/*z* 321.2433 [M + H]^+^ (calcd. for C_20_H_33_O_3_, 321.2435; Δ 0.68 ppm).

### 3.4. RSV Antiviral Assay

The procedure was performed as previously described [7].

### 3.5. X-Ray Diffraction

The X-ray diffraction data of **5** were measured on a Bruker D8 Venture PHOTON II CMOS diffractometer equipped with a Cu Kα INCOATEC ImuS micro-focus source (λ = 1.54178 Å). Indexing was performed using APEX4 (Difference Vectors method) [11]. Data integration and reduction were performed using SaintPlus [12]. Absorption correction was performed with the multi-scan method implemented in SADABS [13]. Space group was determined using XPREP implemented in APEX3 [14]. Structure was solved using SHELXT and refined using SHELXL-2019/1 (full-matrix least squares on F2) through the OLEX2 interface program [15,16]. The ellipsoid plot was made with Olex2 [16]. Hydrogen atoms of -OH group and H_2_O molecules were freely refined.

## 4. Conclusions

Herein, we reported five unreported sesterterpenes during our ongoing investigation of the chemical diversity of Antarctica and the sponge *Suberites* sp. Three of the metabolites displayed an isolated phenol ring (**2**–**4**) with abeosuberitandiol (**3**), showing ring contraction, and furanosuberitandiol (**4**) displayed a new dihydrofuran ring. The biological activity of the unreported metabolites was tested together with the known suberitenone compounds A–D (**6**–**9**) and oxaspirosuberitenone (**10**). Notably, **6**, **7**, and **9** displayed interesting activity against respiratory syncytial virus (RSV). This research highlights the importance of marine organisms and their potential in drug discovery and supports the further investigation of relatively unexplored polar habitats.

## Figures and Tables

**Table 1:** NMR data for **1** (600 (^1^H) and 150 (^13^C) MHz, MeOD).

Position	δ_C_, Type	δ_H_ (mult., (*J*))	gCOSY	gHMBC
**1**	65.2, CH	4.25, dd (4.5, 5.0)	2, 6	2, 3, 5
**2**	145.3, CH	6.80, dq (1.3, 5.6)	1, 21	1, 4, 6, 21
**3**	137.2, C			
**4**	202.2 *, C			
**5a**	37.6, C	2.75, dd (13.5, 16.1)	5b, 6	1, 4, 6, 7
**5b**		2.15, dd (3.5, 16.1)	5a, 6	1, 3, 4, 6, 7
**6**	38.5, CH	3.35, o/l*	1, 5a, 5b	1, 5, 7, 22
**7**	134.2, C			
**8**	202.1 *, C			
**9a**	35.6, CH_2_	2.52, d (12.6)	9b, 10	8, 10, 11
**9b**		2.50, d (4.9)	9a, 10	
**10**	54.9, CH	1.96, dd (5.4, 12.5)	9a, 9b	8, 9, 11, 22, 23, 24
**11**	37.7, C			
**12a**	43.9, CH_2_	2.06, o/l *	12b, 13	10, 11, 13, 14, 22, 23
**12b**		1.66, o/l *	12a, 13	11, 22, 23
**13**	72.0, CH	5.59, ddd (2.6, 2.8, 2.9)	12a, 12b, 14	12, 14, 26
**14**	57.3, CH	1.23, o/l *	13	15, 19, 20, 24
**15**	38.2, C			
**16a**	42.2, CH_2_	1.69, o/l *	16b, 17a, 17b	15
**16b**		0.98, o/l *	16a, 17a, 17b	
**17a**	19.5, CH_2_	1.81, o/l *	16a, 16b, 17b, 18a, 18b	
**17b**		1.51, qd (3.1, 14.3)	16a, 16b, 17a, 18a, 18b	15, 19
**18a**	45.3, CH_2_	1.41, ddd (3.5, 4.0, 12.9)	17a, 17b, 18b	16
**18b**		1.26, o/l *	17a, 17b, 18a	
**19**	35.1, C			
**20**	33.3, CH_3_	0.94, s		14, 18, 19, 25
**21**	15.7, CH_3_	1.79, s		2, 3, 4
**22**	160.7, C	6.40, s		6, 7, 8, 10, 12, 23
**23**	20.9, CH_3_	1.29, s		10, 11, 12, 22
**24**	17.4, CH_3_	1.34, s		10, 14, 15, 16
**25**	23.7, CH_3_	1.06, s		14, 18, 19, 20
**26**	172.1, C			
**27**	21.9, CH_3_	2.07, s		26

* Overlapping ^1^H and ^13^C NMR signals; 2D assignments based on proximity likelihood.

**Table 2:** ^1^H and ^13^C NMR data for **2–4** (400 (^1^H) and 100 (^13^C) MHz, CDCl_3_).

Position	2		3		4	
	δ_C_, Type	δ_H_ (mult., (*J*))	δ_C_, Type	δ_H_ (mult., (*J*))	δ_C_, Type	δ_H_ (mult., (*J*))
**1**	116.6, CH	6.90, d (7.8)	121.4, CH	7.43, dd (1.0, 7.9)	155.3, C	
**2**	130.7, CH	7.07, d (7.9)	131.0, CH	7.19, d (7.8)	112.1, CH	6.70, s
**3**	122.0, C		130.6, C		128.4, C	
**4**	153.6, C		154.2, C		148.4, C	
**5**	111.4, CH	6.94, s	114.5, CH	7.41, d (1.0)	109.7, CH	6.42, s
**6**	149.5, C		136.0, C		124.2, C	
**7**	74.4, C		50.1, CH	3.58, ddd (4.0, 7.9, 11.9)	83.3, C	
**8**	41.1, CH_2_	1.90, o/l *	202.1, C		210.8, C	
**9a**	17.5, CH_2_	1.86, o/l *	26.2, CH_2_	2.09, o/l *	33.9, CH_2_	2.48, dd (10.6, 19.0)
**9b**		1.69, o/l *		1.66, ddd (3.0, 3.5, 12.8)		2.33, dd (19.1, 9.0)
**10**	58.9, CH	1.06, o/l *	56.4, CH	1.30, dd (7.1, 12.9)	45.9, CH	1.88, t (9.6)
**11**	34.8, C		43.1, C		38.3, C	
**12a**	46.7, CH_2_	1.92, o/l *	42.6, CH_2_	2.18, dd (2.1, 14.5)	38.9, CH_2_	2.29, dd (4.2, 15.1)
**12b**		1.27, o/l *		1.39, o/l *		1.82, dd (2.4, 15.0)
**13**	70.6, CH	5.49, dt (2.5, 3.1)	70.7, CH	5.51, br dt (2.4, 2.5)	69.8, CH	5.64, td (2.1, 3.7)
**14**	56.8, CH	1.08, d (2.1)	57.5, CH	1.01, o/l *	55.2, CH	1.04, d (1.6)
**15**	37.2, C		36.9, C		37.4, C	
**16a**	41.9, CH_2_	1.82, o/l *	41.5, CH_2_	1.40, o/l *	41.8, CH_2_	1.37, o/l *
**16b**		0.92, o/l *		0.94, o/l *		0.76, dt (2.8, 12.2)
**17a**	18.6, CH_2_	1.75, o/l *	18.1, CH_2_	1.70, o/l *	18.1, CH_2_	1.62, m
**17b**		1.50, o/l *		1.41, o/l *		1.37, o/l *
**18a**	44.3, CH_2_	1.39, o/l *	44.3, CH_2_	1.39, o/l *	43.6, CH_2_	1.36, o/l *
**18b**		1.20, o/l *		1.20, o/l *		1.17, dd (2.5, 13.7)
**19**	34.1, C		33.7, C		33.8, C	
**20**	32.9, CH_3_	0.94, s	33.0, CH_3_	0.92, s	33.1, CH_3_	0.98, s
**21**	15.3, CH_3_	2.23, s	16.1, CH_3_	2.30, s	16.6, CH_3_	2.23, s
**22a**	57.2, CH_2_	1.59, d (14.3)	83.5, CH	4.02, d (7.8)	97.4, CH	4.19, s
**22b**		1.51, o/l *				
**23**	22.7, CH_3_	1.43, s	14.2, CH_3_	1.16, s	18.9, CH_3_	1.00, s
**24**	17.3, CH_3_	1.25, s	16.7, CH_3_	1.26, s	16.3, CH_3_	1.26, s
**25**	23.0, CH_3_	1.03, s	22.9, CH_3_	1.02, s	23.2, CH_3_	1.00, s
**26**	170.6, C		170.4, C		170.2, C	
**27**	21.9, CH_3_	2.07, s	21.8, CH_3_	2.08, s	21.8, CH_3_	2.06, s

* Overlapping ^1^H NMR signals; 2D assignments based on proximity likelihood.

**Table 3:** RSV antiviral (IC_50_) and A549 cytotoxicity (IC_50_) activities.

Compound	Antiviral IC_50_ (μM) ± SD	Cytotoxicity IC_50_ (μM) ± SD
**1**	39.8 ± 2.7	NA *
**2**	>50 ± ND ^†^	ND
**3**	>50 ± ND	ND
**4**	>50 ± ND	ND
**5**	>50 ± ND	ND
**9**	15.0 ± 1.0	35.8 ± 1.2

* Not active at highest concentration tested (107 μM); ^†^ Not determined due to lack of antiviral activity.

## Data Availability

The NMR data for the following compounds have been deposited in the Natural Products Magnetic Resonance Database (NP-MRD; www.np-mrd.org) and can be found under entries NP0341897 (suberitenone K), NP0341898 (suberitandiol), NP0341899 (abeosuberitandiol), NP0341900 (furanosuberitandiol), and NP0341901 (norsuberitenone B). X-ray metadata have been deposited at the Cambridge Crystallographic Data Centre, deposition number 2377382. Other data not found in the Supporting Information will be available upon request to the corresponding author.

## Supplementary Materials

The following supporting information can be downloaded at: https://www.mdpi.com/article/10.3390/md22120551/s1, ^1^H, ^13^C, COSY, HSQC, HMBC, and NOESY NMR spectra and HR-MS of **1**–**5**.
