# Supplementary material for: New Sesterterpenes from the Antarctic Sponge Suberites sp
Source: Mar Drugs. 2024 Dec 6;22(12):551. doi: 10.3390/md22120551 (PMC11676953; doi:10.3390/md22120551)
Supplement: Supplementary file 1 [file marinedrugs-22-00551-s001.zip › marinedrugs-3318565-supplementary.pdf]

New Sesterterpenes from the Antarctic Sponge *Suberites* sp.

Stine S. H. Olsen[1], Sydney K. Morrow [1], Julia L. Szabo [1], Michael N. Teng [2], Kim C. Tran [2], Charles D. Amsler [3], James B. McClintock [3] and Bill. J. Baker [1,]*

[1]Department of Chemistry, University of South Florida, 4202 E. Fowler Avenue, CHE205, Tampa, FL 33620, USA

[2]Department of Internal Medicine, University of South Florida, Tampa, FL, 33612, USA

[3]Department of Biology, University of Alabama at Birmingham, 1300 University Blvd, Birmingham, AL 35233, USA

Table of Contents

Table S1 – NMR data for suberitedione (**1**) (600 (^{1}H) and 150 (^{13}C) MHz, MeOD). 3
Figure S01 – Structure of **1** 4
Figure S02 – ^{1}H NMR spectrum (600 MHz, MeOD) of **1** 4
Figure S03 – ^{1}H NMR spectrum zoomed (600 MHz, MeOD) of **1** 5
Figure S04 – ^{13}C NMR spectrum (150 MHz, MeOD) of **1** 5
Figure S05 – ^{13}C NMR spectrum zoomed (150 MHz, MeOD) of **1** 6
Figure S06 – COSY NMR spectrum (600 MHz, MeOD) of **1** 6
Figure S07 – HSQC NMR spectrum (600 MHz, MeOD) of **1** 7
Figure S08 – HMBC NMR spectrum (600 MHz, MeOD) of **1** 7
Figure S09 – NOESY NMR spectrum (600 MHz, MeOD) of **1** 8
Figure S10 – HRESIMS analysis of **1** 8
Table S2 – NMR data for suberiphenol B (**2**) (400 (^{1}H) and 100 (^{13}C) MHz, ^{a}CDCl$_3$, ^{b}(CD$_3$)$_2$SO). 9
Figure S11 – Chemical structure of **2** 10
Figure S12 – ^{1}H NMR spectrum (400 MHz, CDCl$_3$) of **2** 10
Figure S13 – ^{13}C NMR spectrum (100 MHz, CDCl$_3$) of **2** 11
Figure S14 – ^{13}C NMR spectrum zoomed (100 MHz, CDCl$_3$) of **2** 11
Figure S15 – COSY NMR spectrum (400 MHz, CDCl$_3$) of **2** 12
Figure S16 – HSQC NMR spectrum (400 MHz, CDCl$_3$) of **2** 12
Figure S17 – HMBC NMR spectrum (400 MHz, CDCl$_3$) of **2** 13
Figure S18 – NOESY NMR spectrum (400 MHz, CDCl$_3$) of **2** 13
Figure S19 – ^{1}H NMR spectrum (400 MHz, (CD$_3$)$_2$SO) of **2** 14
Figure S20 – HSQC NMR spectrum (400 MHz, (CD$_3$)$_2$SO) of **2** 14
Figure S21 – NOESY NMR spectrum (400 MHz, (CD$_3$)$_2$SO) of **2** 15
Figure S22 – HRESIMS analysis of **2** 15
Table S3 – NMR data for suberiphenol C (**3**) (400 (^{1}H) and 100 (^{13}C) MHz, CDCl$_3$)................ 16
Figure S23 – Chemical structure of **3** 17
Figure S24 – ^{1}H NMR spectrum (400 MHz, CDCl$_3$) of **3** 17
Figure S25 – ^{13}C NMR spectrum (100 MHz, CDCl$_3$) of **3** 18
Figure S26 – ^{13}C NMR spectrum zoomed (100 MHz, CDCl$_3$) of **3** 18
Figure S27 – COSY NMR spectrum (400 MHz, CDCl$_3$) of **3** 19

Figure S28 – HSQC NMR spectrum (400 MHz, $CDCl_3$) of **3** 19
Figure S29 – HMBC NMR spectrum (400 MHz, $CDCl_3$) of **3** 20
Figure S30 – NOESY NMR spectrum (400 MHz, $CDCl_3$) of **3** 20
Figure S31 – HRESIMS analysis of **3** 21
Table S4 – NMR data for suberiphenol D (**4**) (400 (^{1}H) and 100 (^{13}C) MHz, ^{a}$CDCl_3$, ^{b}$(CD_3)_2SO$). 22
Figure S32 – Structure of **4** 23
Figure S33 – ^{1}H NMR spectrum (400 MHz, $CDCl_3$) of **4** 23
Figure S34 – ^{13}C NMR spectrum (100 MHz, $CDCl_3$) of **4** (δ_C 50.9, MeOH contaminant) 24
Figure S35 – ^{13}C NMR spectrum zoomed (100 MHz, $CDCl_3$) of **4** (δ_C 50.9, MeOH contaminant) 24
Figure S36 – COSY NMR spectrum (400 MHz, $CDCl_3$) of **4** 25
Figure S37 – HSQC NMR spectrum (400 MHz, $CDCl_3$) of **4** 25
Figure S38 – HMBC NMR spectrum (400 MHz, $CDCl_3$) of **4** 26
Figure S39 – NOESY NMR spectrum (400 MHz, $CDCl_3$) of **4** 26
Figure S40 – ^{1}H NMR spectrum (400 MHz, $(CD_3)_2SO$) of **4** 27
Figure S41 – HSQC NMR spectrum (400 MHz, $(CD_3)_2SO$) of **4** 27
Figure S42 – NOESY NMR spectrum (400 MHz, $(CD_3)_2SO$) of **4** 28
Figure S43 – HRESIMS analysis of **4** 28
Table S5 – NMR data for suberone (**5**) (400 (^{1}H) and 100 (^{13}C) MHz, $CDCl_3$). 29
Figure S44 – Structure of **5** 30
Figure S45 – ^{1}H NMR spectrum (400 MHz, $CDCl_3$) of **5** 30
Figure S46 – ^{13}C NMR spectrum (100 MHz, $CDCl_3$) of **5** 31
Figure S47 – COSY NMR spectrum (400 MHz, $CDCl_3$) of **5** 31
Figure S48 – HSQC NMR spectrum (400 MHz, $CDCl_3$) of **5** 32
Figure S49 – HMBC NMR spectrum (400 MHz, $CDCl_3$) of **5** 32
Figure S50 – NOESY NMR spectrum (400 MHz, $CDCl_3$) of **5** 33
Figure S51 – HRESIMS analysis of **5** 33
Table S6 – Crystal Data and Structure Refinement for Suberone (**5**) 34
Figure S52 – Ellipsoid plot of **5**. Anisotropic displacement parameters were drawn at 50% probability level. 35
Figure S53 – RSV Transcription vs [treatment] curve for suberitenone K (**1**) and suberitenone D (**9**) 36
Figure S54 – A549 Cell viability vs [treatment] curve for suberitenone D (**9**).. 36

Table S1 – NMR data for suberitedione (**1**) (600 (^{1}H) and 150 (^{13}C) MHz, MeOD).

pos	δ_C, type	δ_H (*J* in Hz)	gCOSY	gHMBC	Key NOESY
**1**	65.2, CH	4.25, dd (4.0, 4.0)	2, 6	2, 3, 5	6
**2**	145.3, CH	6.80, dd (5.6, 1.3)	1, 21	1, 4, 6, 21	
**3**	137.2, C				
**4**	202.2*, C				
**5a**	37.6, C	2.75, dd (13.5, 16.1)	5b, 6	1, 4, 6, 7	
**5b**		2.15, dd (16.1, 3.5)	5a, 6	1, 3, 4, 6, 7	
**6**	38.5, CH	3.35, o/l	1, 5a, 5b	1, 5, 7, 22	1
**7**	134.2, C				
**8**	202.1*, C				
**9**	35.6, CH_2	2.51, dd (2.8, 3.2)	10	8, 10, 11	
**10**	54.9, CH	1.96, dd (12.5, 5.4)	9	8, 9, 11, 22, 23, 24	14
**11**	37.7, C				
**12a**	43.9, CH_2	2.06, o/l	12b, 13	10, 11, 13, 14, 22, 23	
**12b**		1.66, o/l	12a, 13	11, 22, 23	
**13**	72.0, CH	5.59, ddd (2.6, 2.8, 2.9)	12a, 12b, 14	12, 14, 26	20
**14**	57.3, CH	1.23, o/l	13	15, 19, 20, 24	10, 20
**15**	38.2, C				
**16a**	42.2, CH_2	1.69, o/l	16b, 17a, 17b	15	
**16b**		0.98, o/l	16a, 17a, 17b		
**17a**	19.5, CH_2	1.81, o/l	16a, 16b, 17b, 18a, 18b		
**17b**		1.51, ddddd (14.3, 3.1, 3.1, 3.1, 3.1)	16a, 16b, 17a, 18a, 18b	15, 19	
**18a**	45.3, CH_2	1.41, ddd (12.9, 4.0, 3.5)	17a, 17b, 18b	16	
**18b**		1.26, o/l	17a, 17b, 18a		
**19**	35.1, C				
**20**	33.3, CH_3	0.94, s		14, 18, 19, 25	13, 14
**21**	15.7, CH_3	1.79, s		2, 3, 4	
**22**	160.7, C	6.40, s		6, 7, 8, 10, 12, 23	
**23**	20.9, CH_3	1.29, s		10, 11, 12, 22	27
**24**	17.4, CH_3	1.34, s		10, 14, 15, 16	25
**25**	23.7, CH_3	1.06, s		14, 18, 19, 20	24
**26**	172.1, C				
**27**	21.9, CH_3	2.07, s		26	23

* Overlapping ^{13}C NMR signals, 2D assignments based on proximity likelihood.

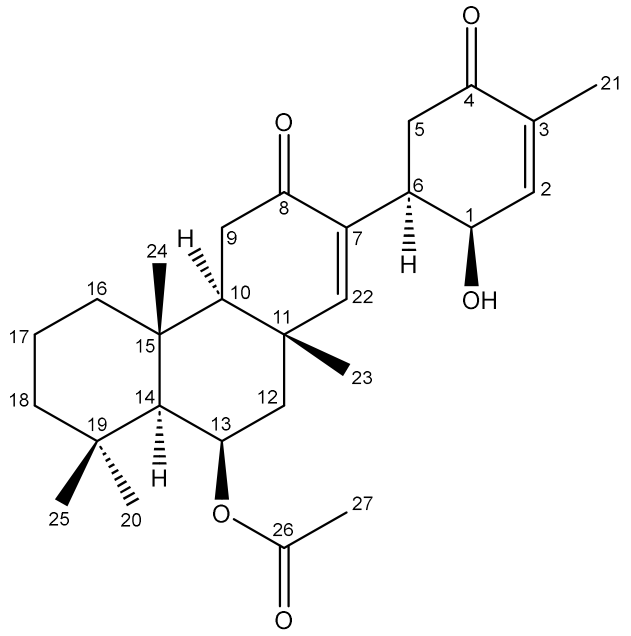

Figure S01 – Structure of **1**

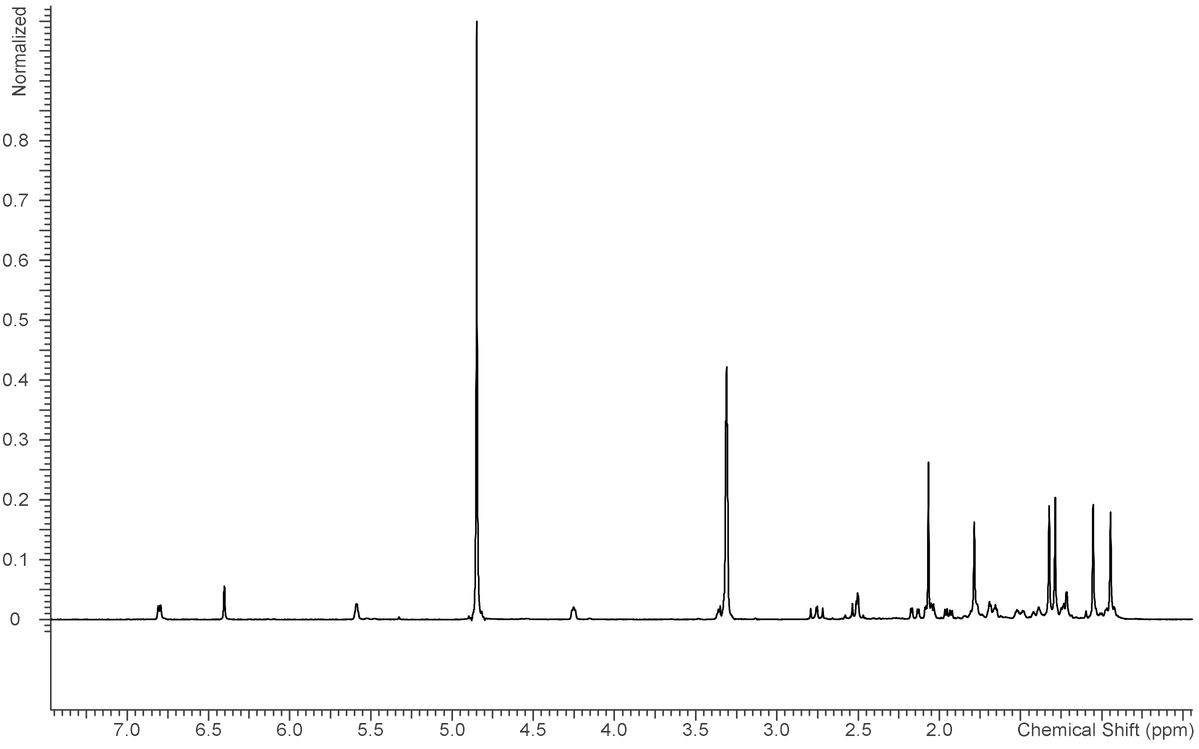

Figure S02 – ^{1}H NMR spectrum (600 MHz, MeOD) of **1**

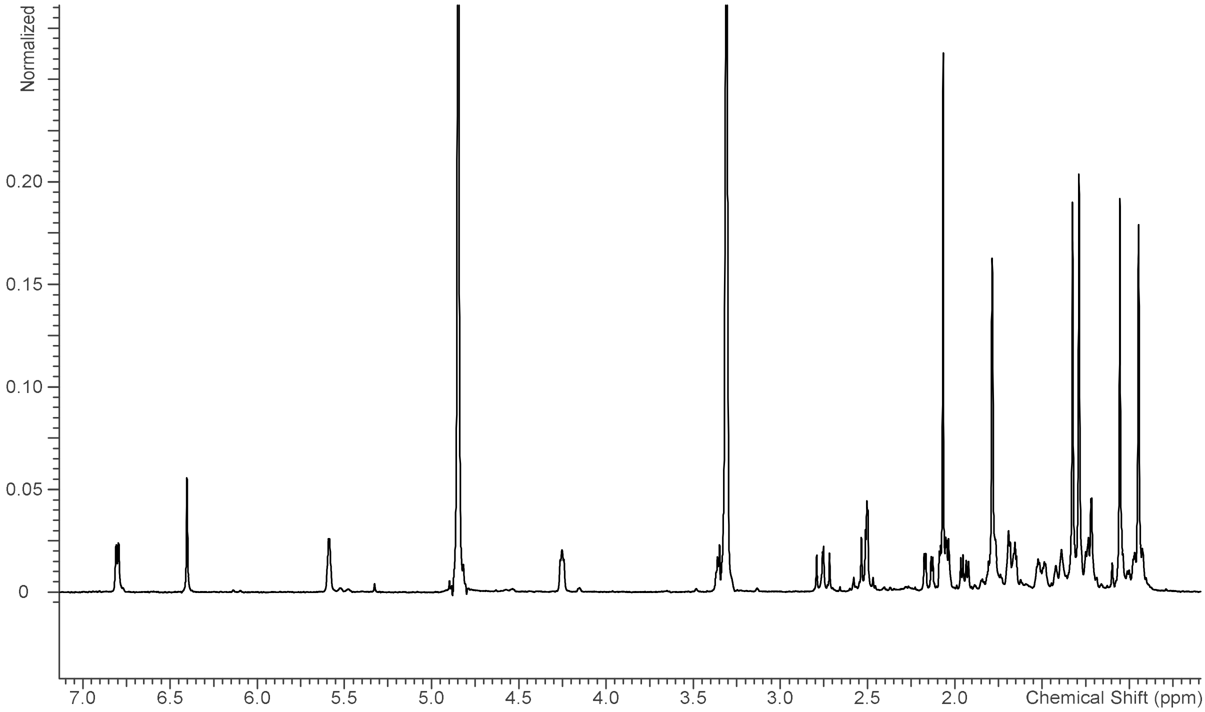

Figure S03 – ^{1}H NMR spectrum zoomed (600 MHz, MeOD) of **1**

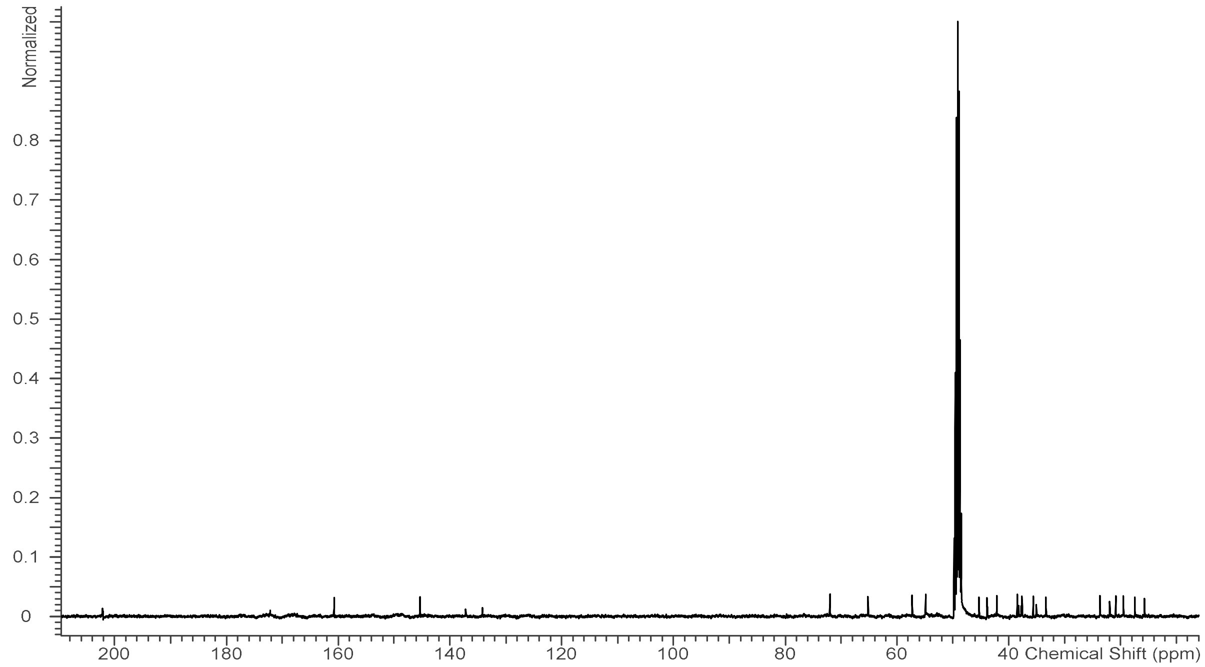

Figure S04 – ^{13}C NMR spectrum (150 MHz, MeOD) of **1**

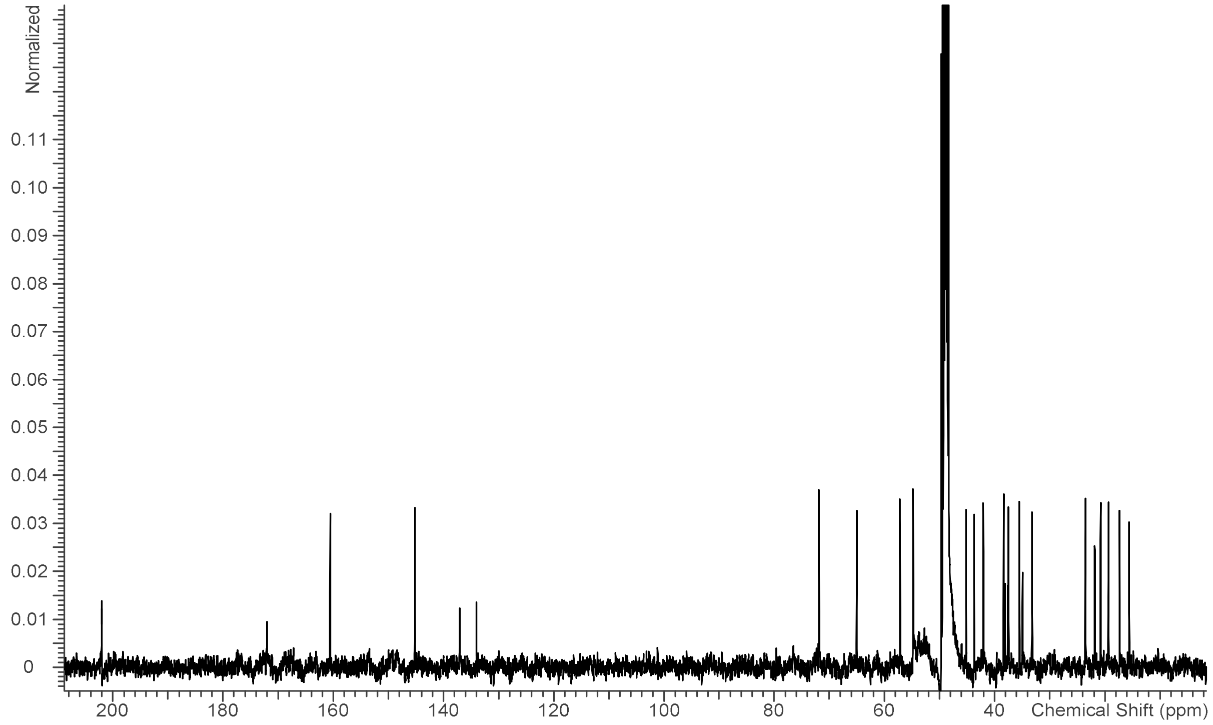

Figure S05 – ^{13}C NMR spectrum zoomed (150 MHz, MeOD) of **1**

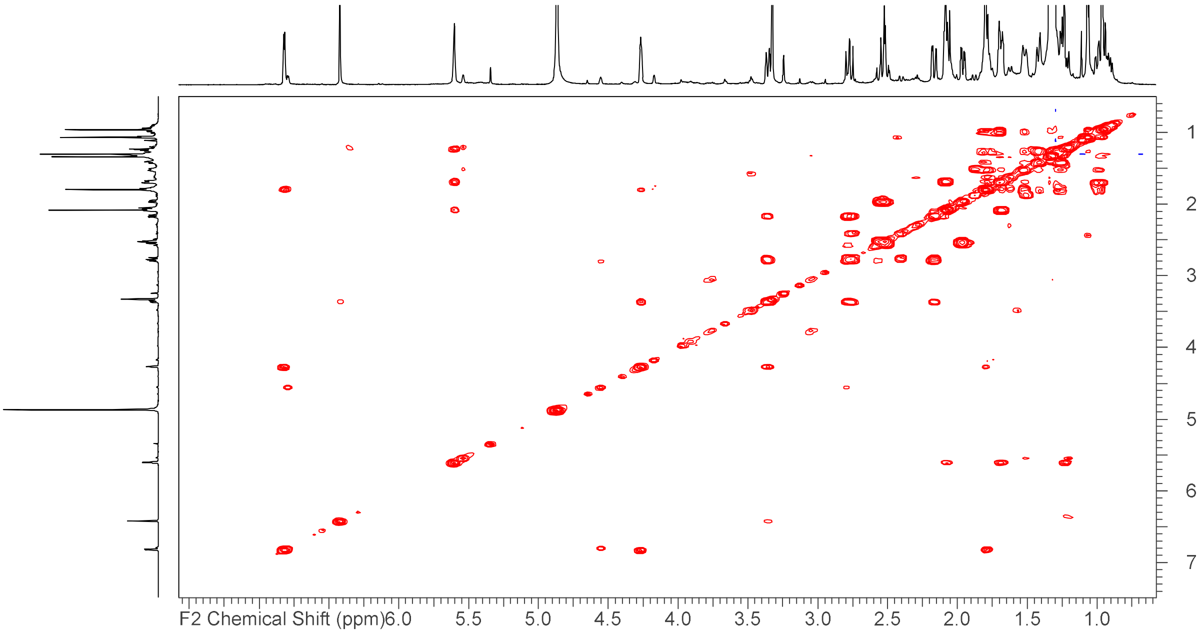

Figure S06 – COSY NMR spectrum (600 MHz, MeOD) of **1**

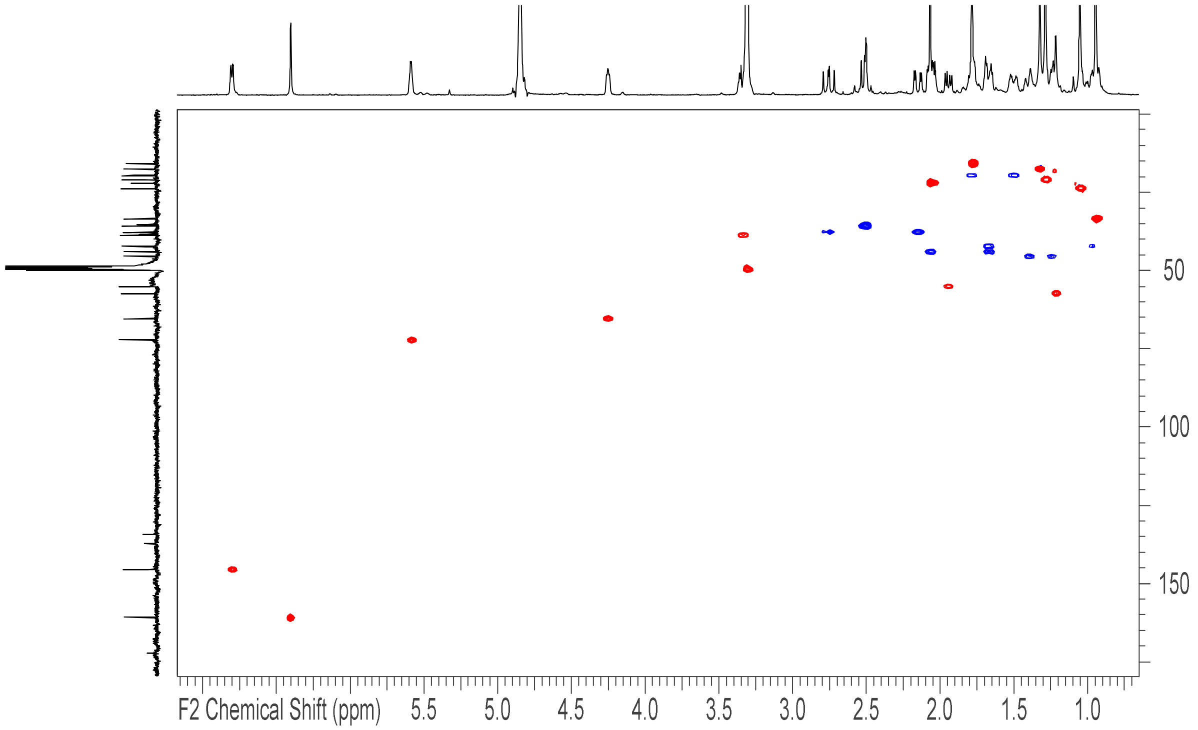

Figure S07 – HSQC NMR spectrum (600 MHz, MeOD) of **1**

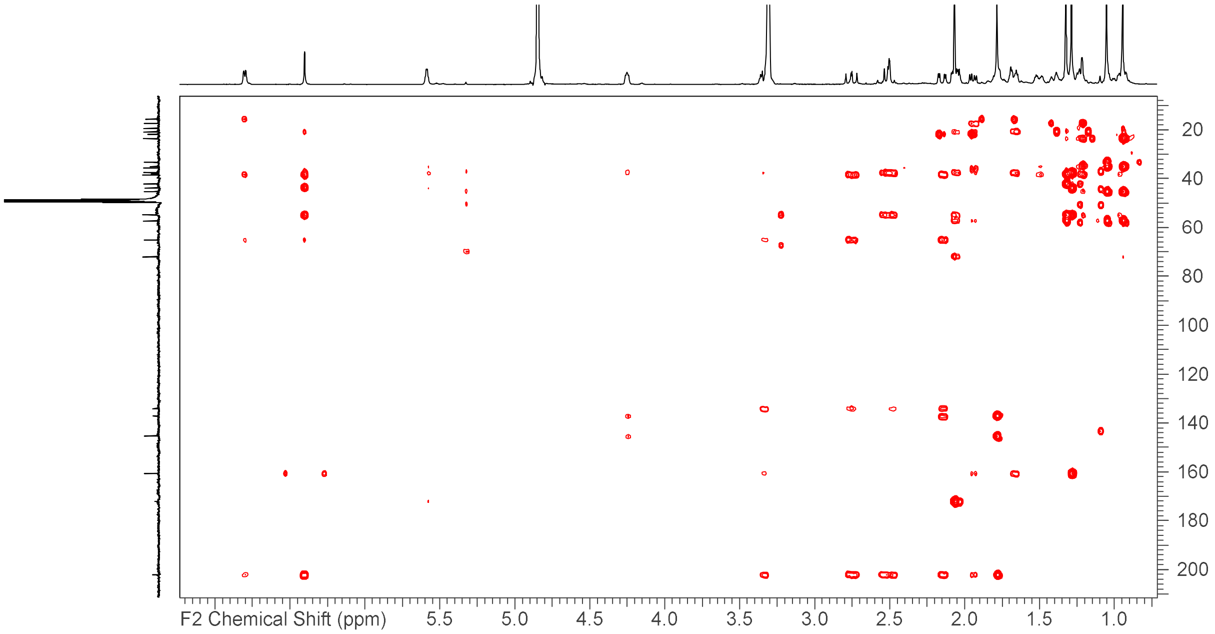

Figure S08 – HMBC NMR spectrum (600 MHz, MeOD) of **1**

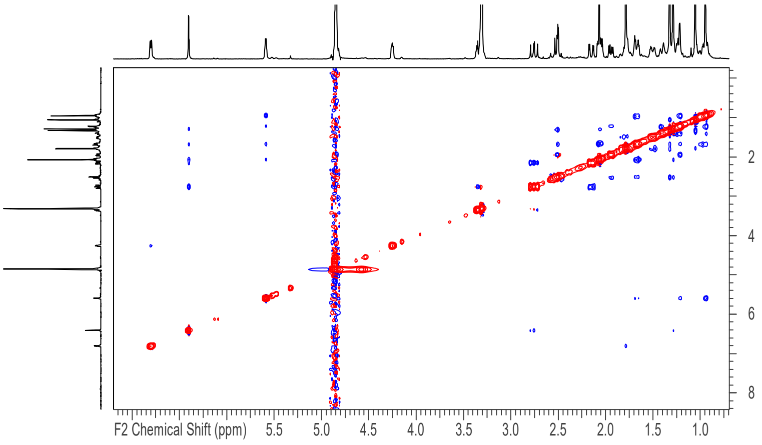

Figure S09 – NOESY NMR spectrum (600 MHz, MeOD) of **1**

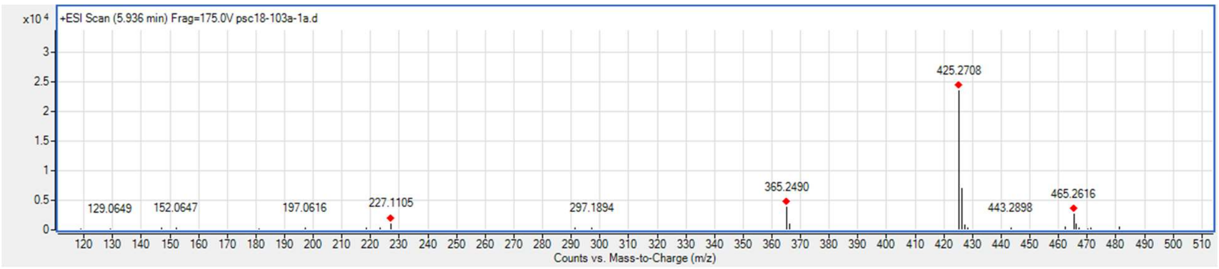

Figure S10 – HRESIMS analysis of **1**

Table S2 – NMR data for suberiphenol B (**2**) (400 (^{1}H) and 100 (^{13}C) MHz, ^{a}CDCl$_3$, ^{b}(CD$_3$)$_2$SO).

pos	$^a\delta_C$, type	$^b\delta_C$, type	$^a\delta_H$ (*J* in Hz)	$^b\delta_H$ (*J* in Hz)	gCOSY	gHMBC	^{a}Key NOESY	^{a}Key NOESY
**1**	116.6, CH	115.7, CH	6.90, d (7.8)	6.72, d (7.8)	2	7		
**2**	130.7, CH	130.0, CH	7.07, d (7.9)	6.92, o/l	1	4, 6, 21		
**3**	122.0, C							
**4**	153.6, C							
**5**	111.4, CH	112.0, CH	6.94, s	6.91, o/l		1, 3, 7		
**6**	149.5, C							
**7**	74.4, C							
**8**	41.1, CH_2	41.1, CH_2	1.90, o/l	1.72, o/l	9a			
**9a**	17.5, CH_2	17.6, CH_2	1.86, o/l	1.73, o/l	8, 10			
**9b**			1.69, o/l	1.51, o/l		7, 10, 11		
**10**	58.9, CH	58.7, CH	1.06, o/l	1.04, o/l	9a		20	20
**11**	34.8, C							
**12a**	46.7, CH_2	47.0, CH_2	1.92, o/l	1.67, o/l	12b	10, 11, 13, 14		
**12b**			1.27, o/l	1.27, o/l	12a, 13			
**13**	70.6, CH	70.3, CH	5.49, ddd (3.1, 3.1, 2.5)	5.33, s	12b, 14		14, 20	14, 20
**14**	56.8, CH	56.1, CH	1.08, d (2.1)	1.11, o/l	13	15, 19, 23	13	13, 20
**15**	37.2, C							
**16a**	41.9, CH_2	41.9, CH_2	1.82, o/l	1.74, o/l	16b	14, 18		
**16b**			0.92, o/l	0.89, o/l	16a			
**17a**	18.6, CH_2	18.7, CH_2	1.75, o/l	1.69, o/l	17b			
**17b**			1.50, o/l	1.43, o/l	17a			
**18a**	44.3, CH_2	44.3, CH_2	1.39, o/l	1.32, o/l	18b			
**18b**			1.20, o/l	1.17, o/l	18a	15, 16,		
**19**	34.1, C							
**20**	32.9, CH_3	32.9, CH_3	0.94, s	0.87, s		14, 18, 19, 25	10, 13	10, 13, 14
**21**	15.3, CH_3	16.1, CH_3	2.23, s	2.05, s		2, 3, 4		
**22a**	57.2, CH_2	56.9, CH_2	1.59, d (14.3)	1.41, o/l	22b	7, 8, 10, 11, 23		
**22b**			1.51, o/l	1.31, o/l	22a	7, 10, 11, 23		
**23**	22.7, CH_3	22.9, CH_3	1.43, s	1.34, s		10, 11, 12, 22		OH-7
**24**	17.3, CH_3	17.6, CH_3	1.25, s	1.18, s		10, 14, 15, 16,	25, 27	
**25**	23.0, CH_3	23.4, CH_3	1.03, s	0.97, s		14, 18, 19, 20	24, 27	
**26**	170.6, C							
**27**	21.9, CH_3	21.9, CH_3	2.07, s	1.99, s		26	23, 24, 25	
**OH-4**			8.97, s					
**OH-7**			4.38, s					23

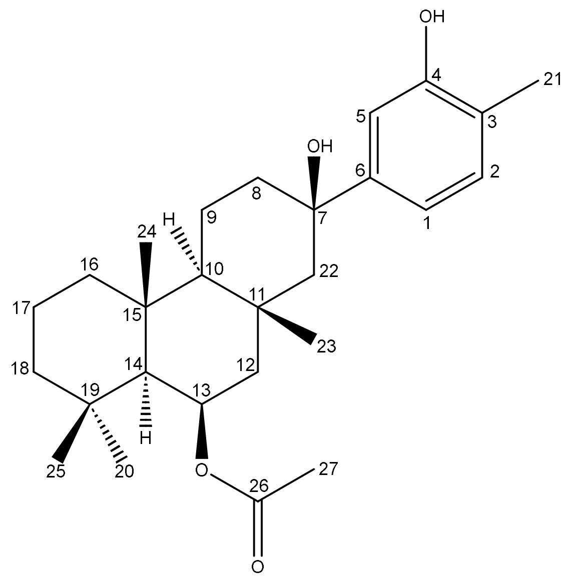

Figure S11 – Chemical structure of **2**

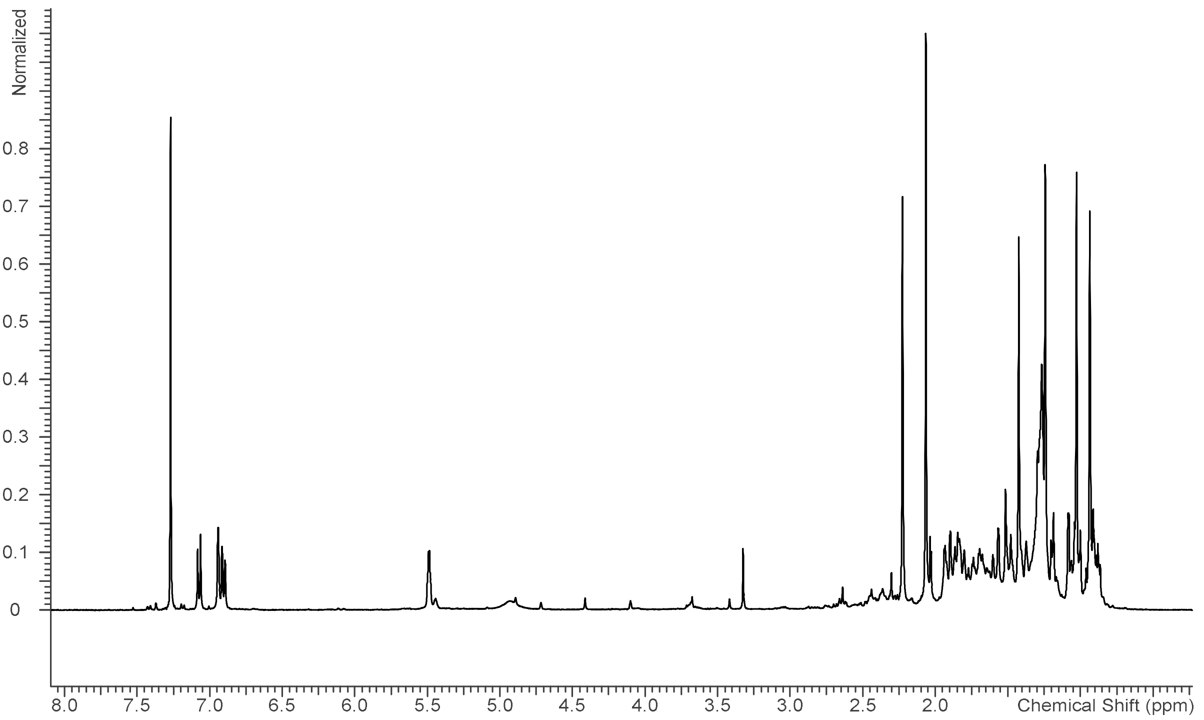

Figure S12 – ^{1}H NMR spectrum (400 MHz, $CDCl_3$) of **2**

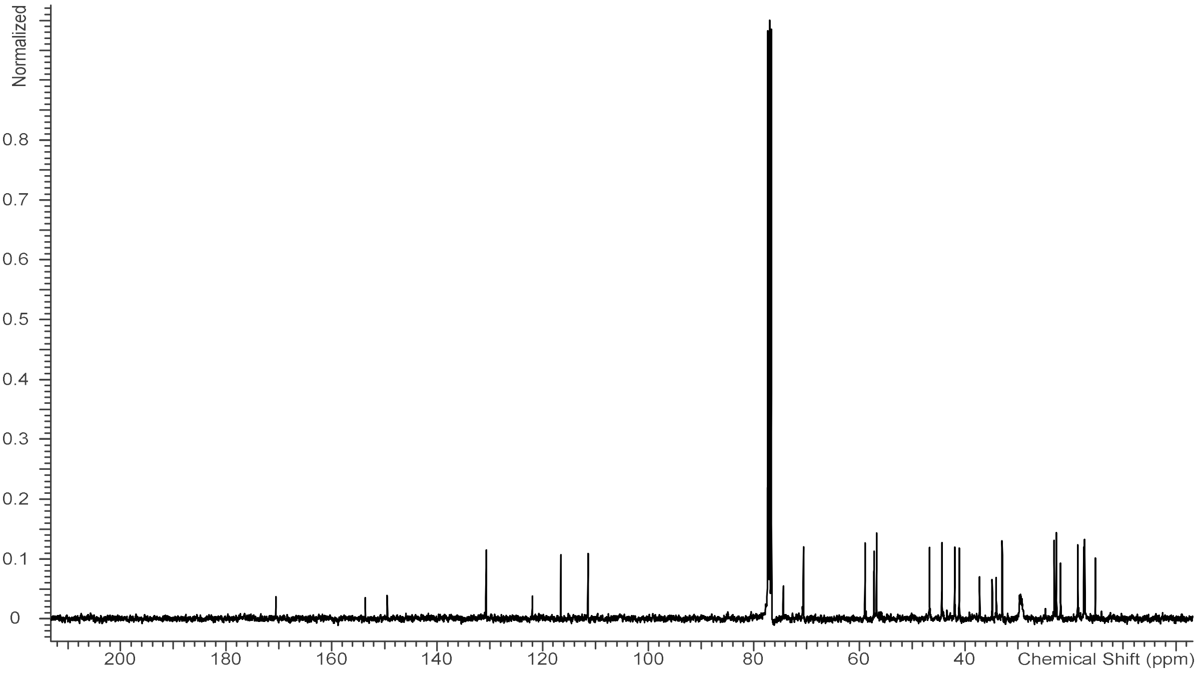

Figure S13 – ^{13}C NMR spectrum (100 MHz, $CDCl_3$) of **2**

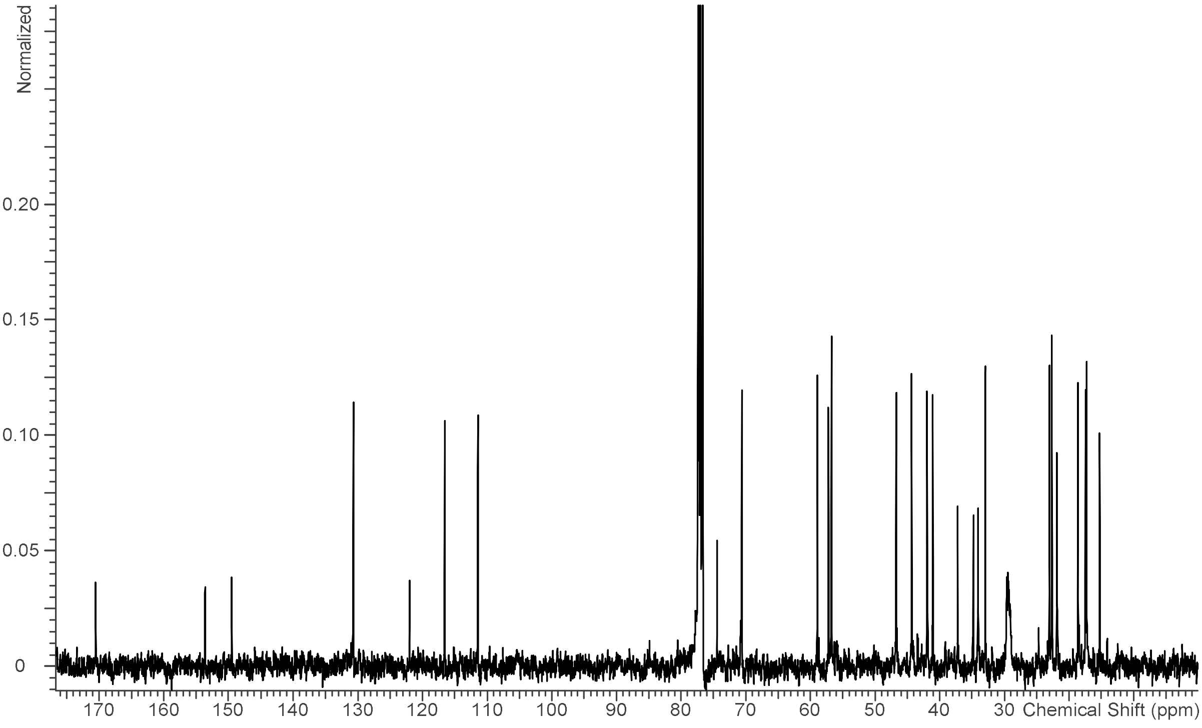

Figure S14 – ^{13}C NMR spectrum zoomed (100 MHz, $CDCl_3$) of **2**

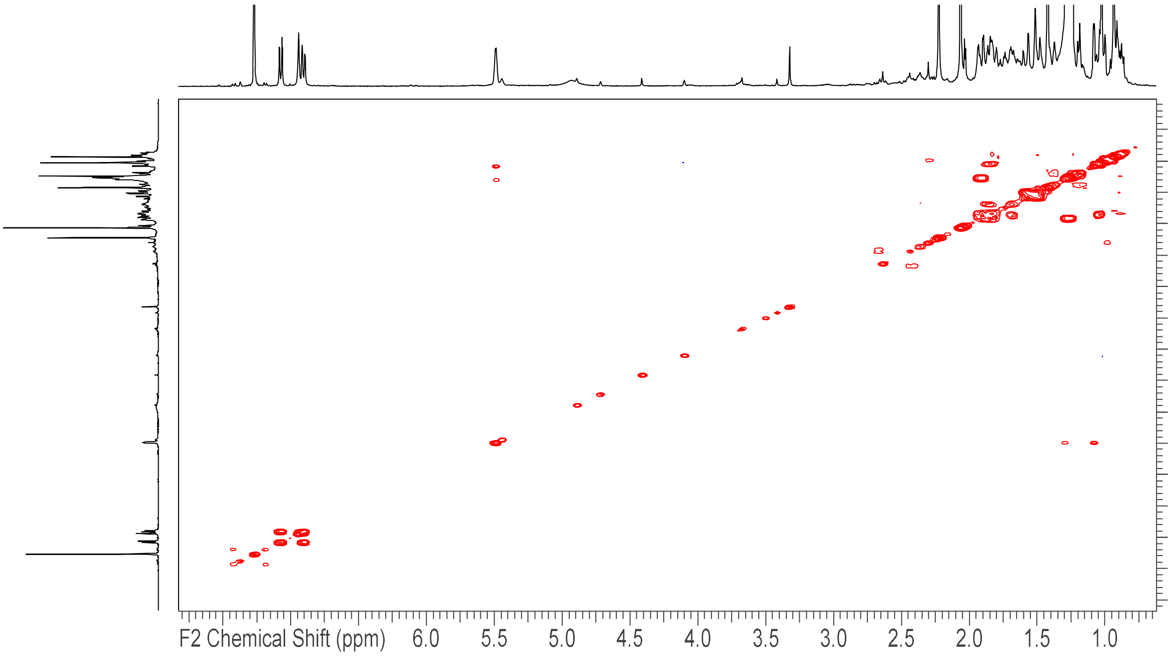

Figure S15 – COSY NMR spectrum (400 MHz, $CDCl_3$) of **2**

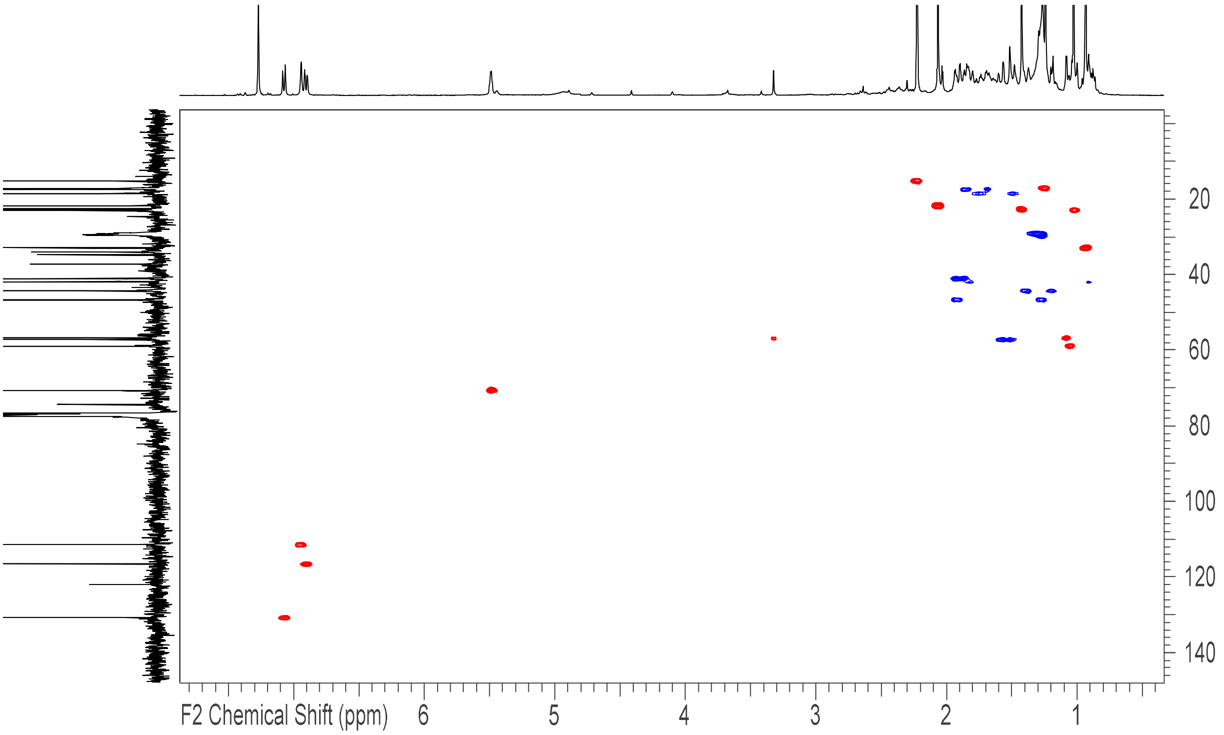

Figure S16 – HSQC NMR spectrum (400 MHz, $CDCl_3$) of **2**

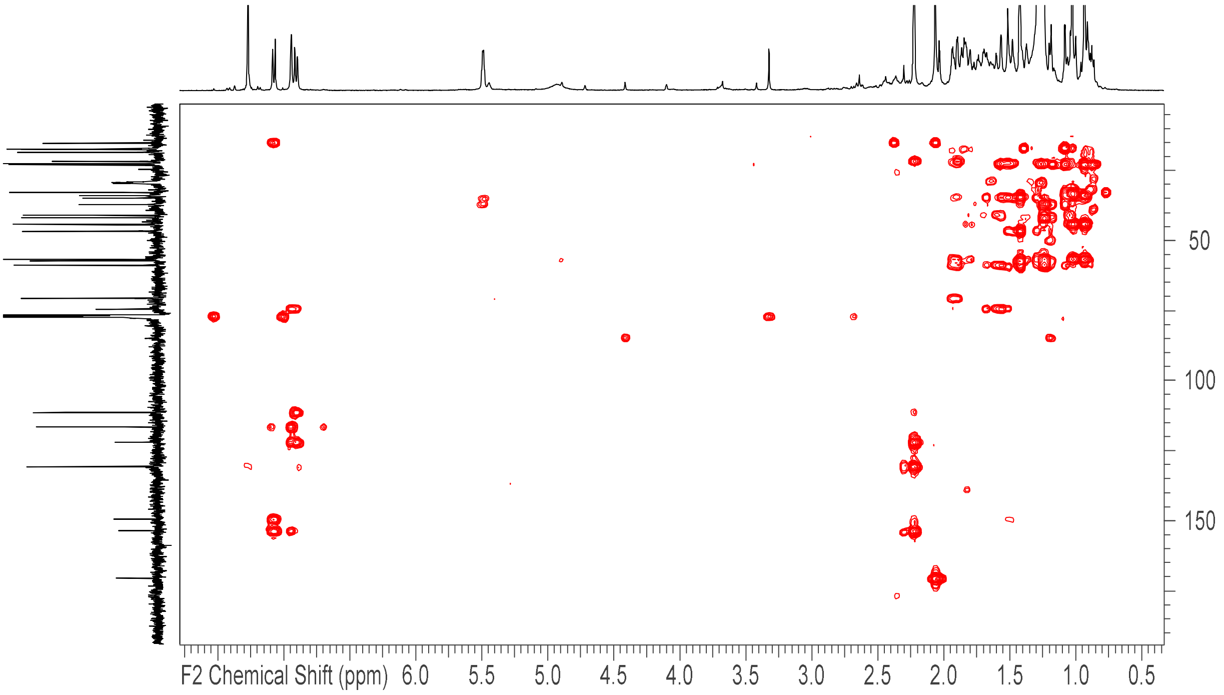

Figure S17 – HMBC NMR spectrum (400 MHz, $CDCl_3$) of **2**

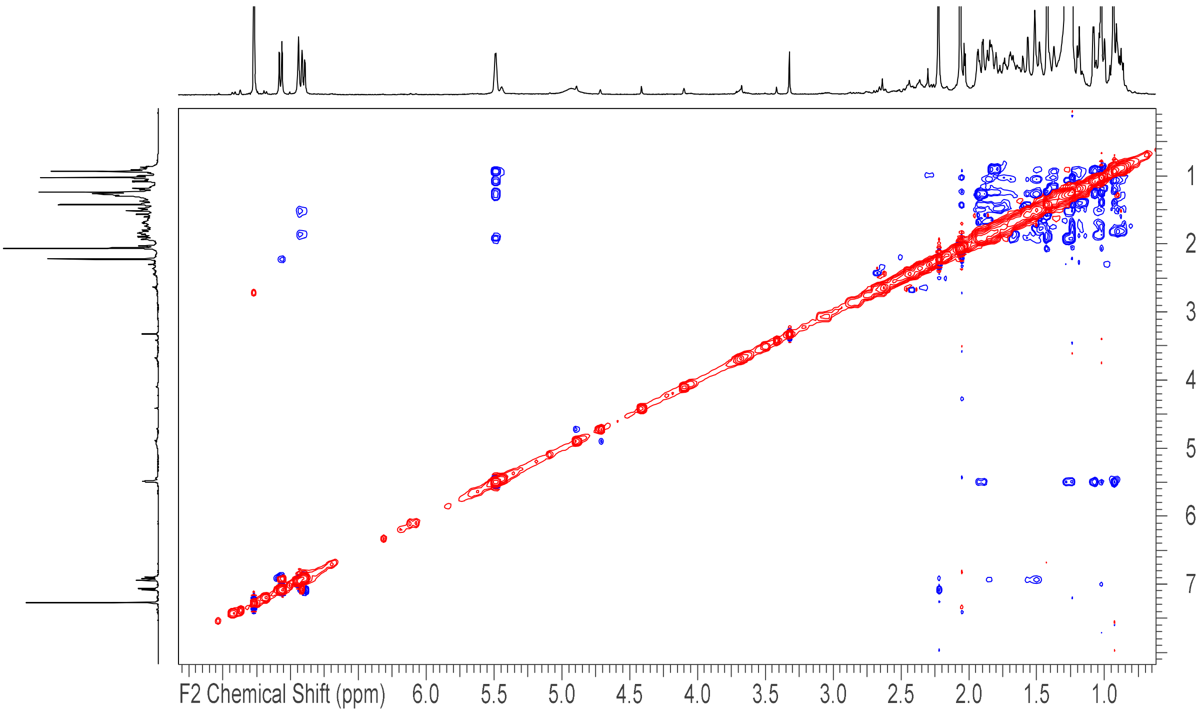

Figure S18 – NOESY NMR spectrum (400 MHz, $CDCl_3$) of **2**

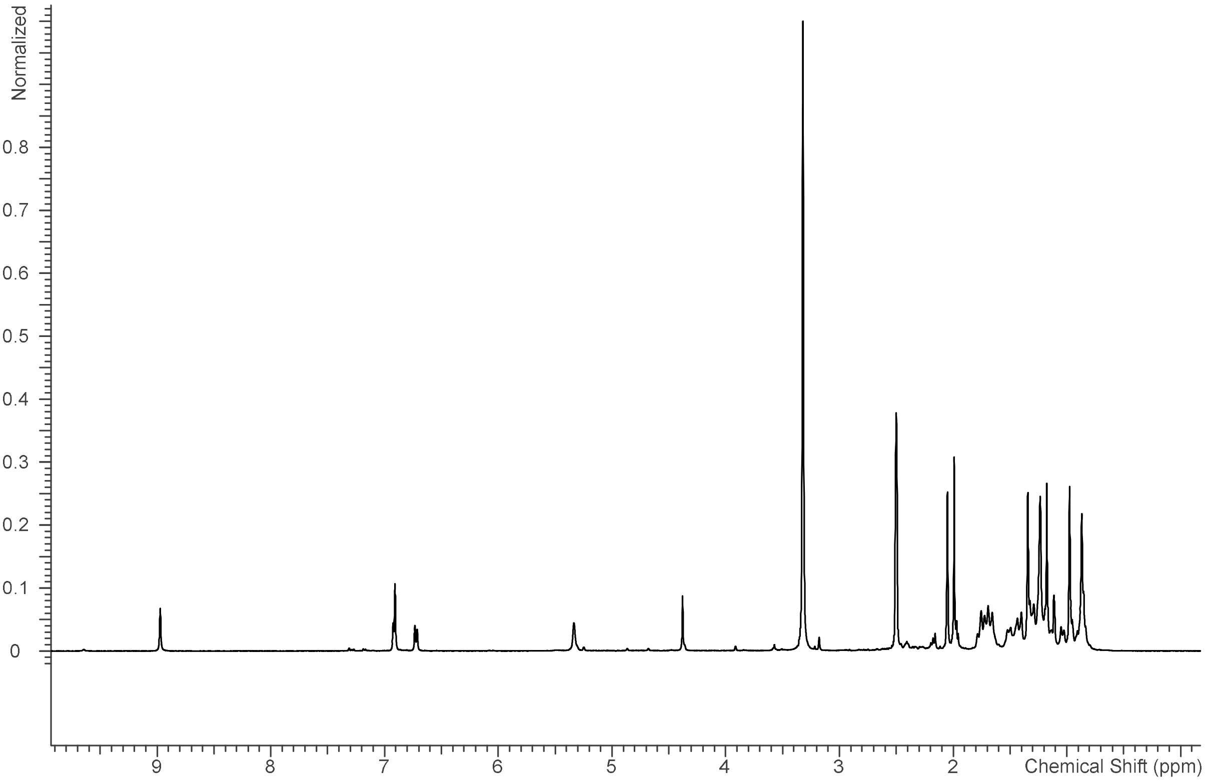

Figure S19 – ^{1}H NMR spectrum (400 MHz, $(CD_3)_2SO$) of **2**

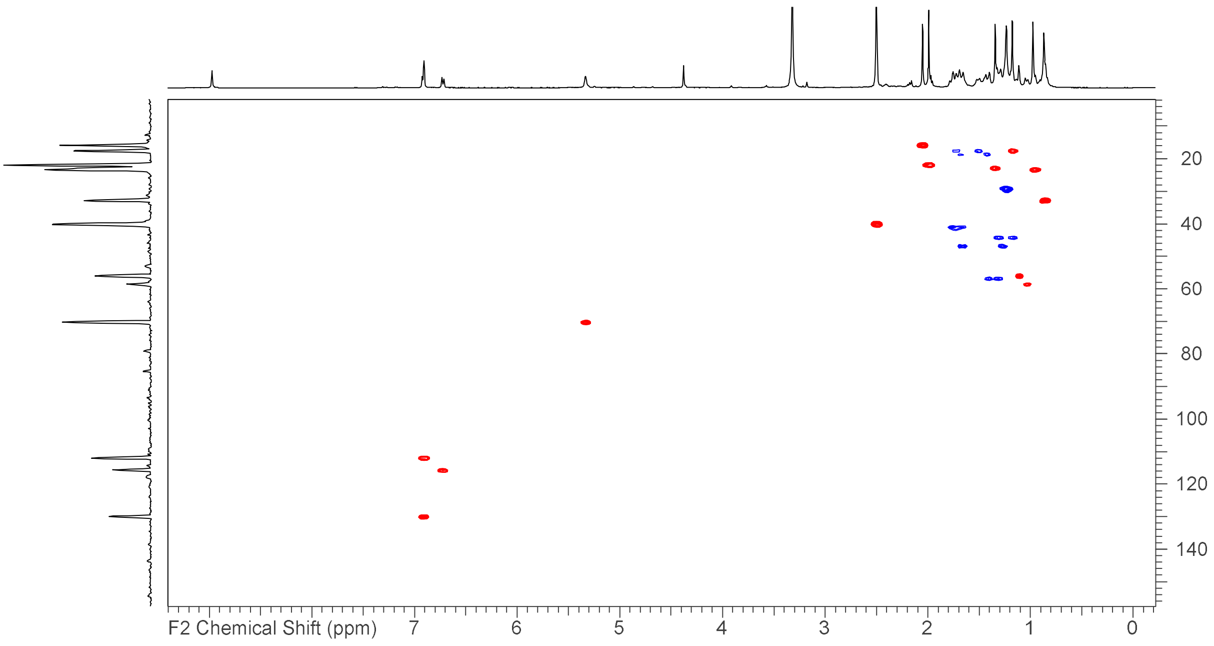

Figure S20 – HSQC NMR spectrum (400 MHz, $(CD_3)_2SO$) of **2**

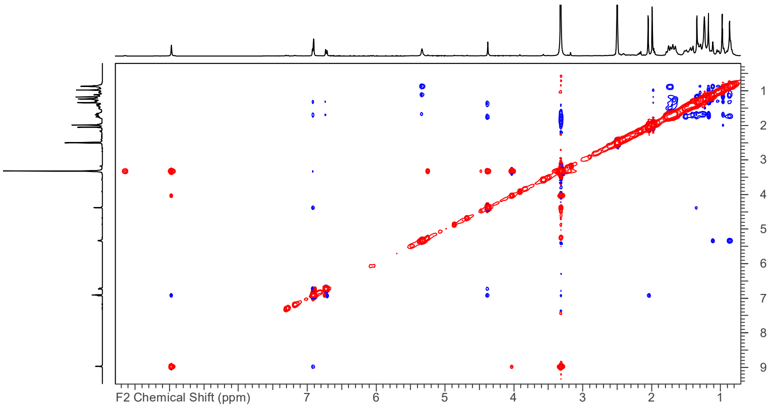

Figure S21 – NOESY NMR spectrum (400 MHz, $(CD_3)_2SO$) of **2**

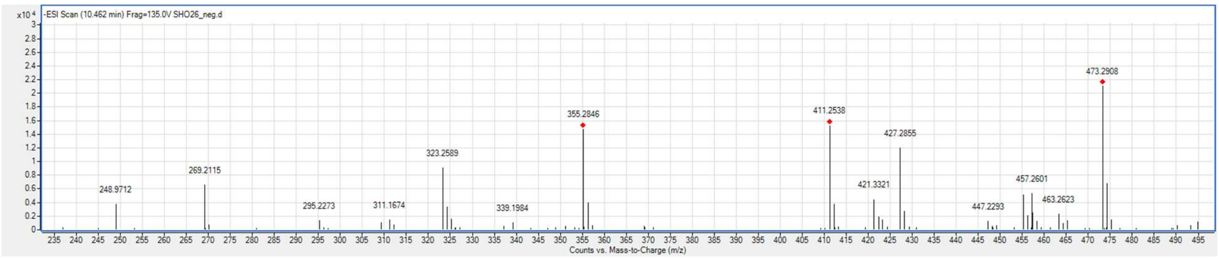

Figure S22 – HRESIMS analysis of **2**

Table S3 – NMR data for suberiphenol C (**3**) (400 (^{1}H) and 100 (^{13}C) MHz, $CDCl_3$).

pos	δ_C, type	δ_H (J in Hz)	gCOSY	gHMBC	Key NOESY
**1**	121.4, CH	7.43, d (7.4)	2	3, 5, 8	
**2**	131.0, CH	7.19, d (7.6)	1, 21	4, 6, 21	
**3**	130.6, C				
**4**	154.2, C				
**5**	114.5, CH	7.41, s		1	
**6**	136.0, C				
**7**	50.1, CH	3.58, ddd (11.9, 7.9, 4.0)	9a, 9b, 22		23
**8**	202.1, C				
**9a**	26.2, CH_2	2.09, o/l	7, 9b, 10	7, 8, 10	
**9b**		1.66, ddd (12.8, 3.5 3.0)	7, 9a, 10	8	
**10**	56.4, CH	1.30, dd (12.9, 7.1)	9a, 9b	21, 23	22
**11**	43.1, C				
**12a**	42.6, CH_2	2.18, dd (14.5, 2.1)	12b	10, 11, 13	
**12b**		1.39, o/l	12a, 13		
**13**	70.7, CH	5.51, br ddd (2.5, 2.5, 2.4)	12b, 14	15	14, 20
**14**	57.5, CH	1.01, o/l	13	24, 25	13
**15**	36.9, C				
**16a**	41.5, CH_2	1.40, o/l	16b		
**16b**		0.94, o/l	16a		
**17a**	18.1, CH_2	1.70, o/l			
**17b**		1.41, o/l		24	
**18a**	44.3, CH_2	1.39, o/l	18b		
**18b**		1.20, o/l	18a	25	
**19**	33.7, C				
**20**	33.0, CH_3	0.92, s		18, 19, 25	13
**21**	16.1, CH_3	2.30, s		3, 4	
**22**	83.5, CH	4.02, d (7.8)	7	8, 12, 23	10
**23**	14.2, CH_3	1.16, s		10, 12, 22	7, 27
**24**	16.7, CH_3	1.26, s		10, 15, 16	25
**25**	22.9, CH_3	1.02, s		18, 19, 20	24, 27
**26**	170.4, C				
**27**	21.8, CH_3	2.08, s		26	23, 25

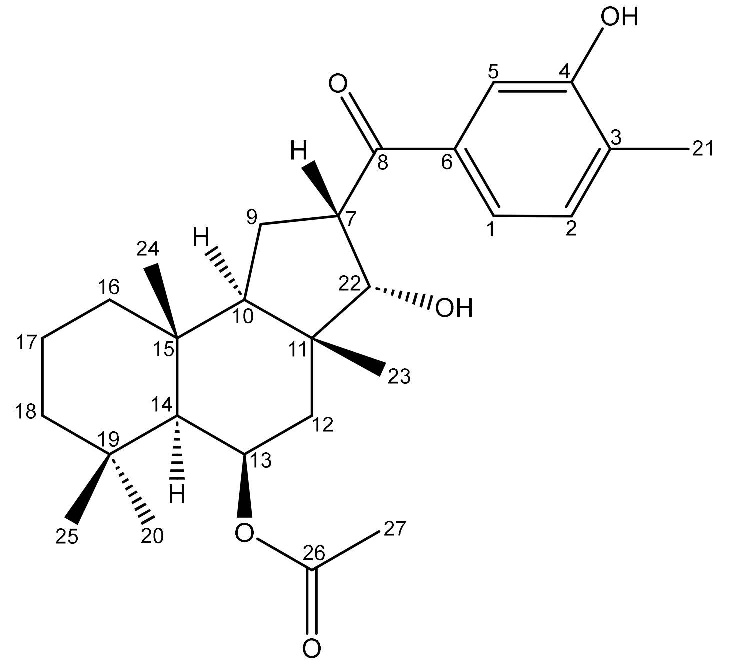

Figure S23 – Chemical structure of **3**

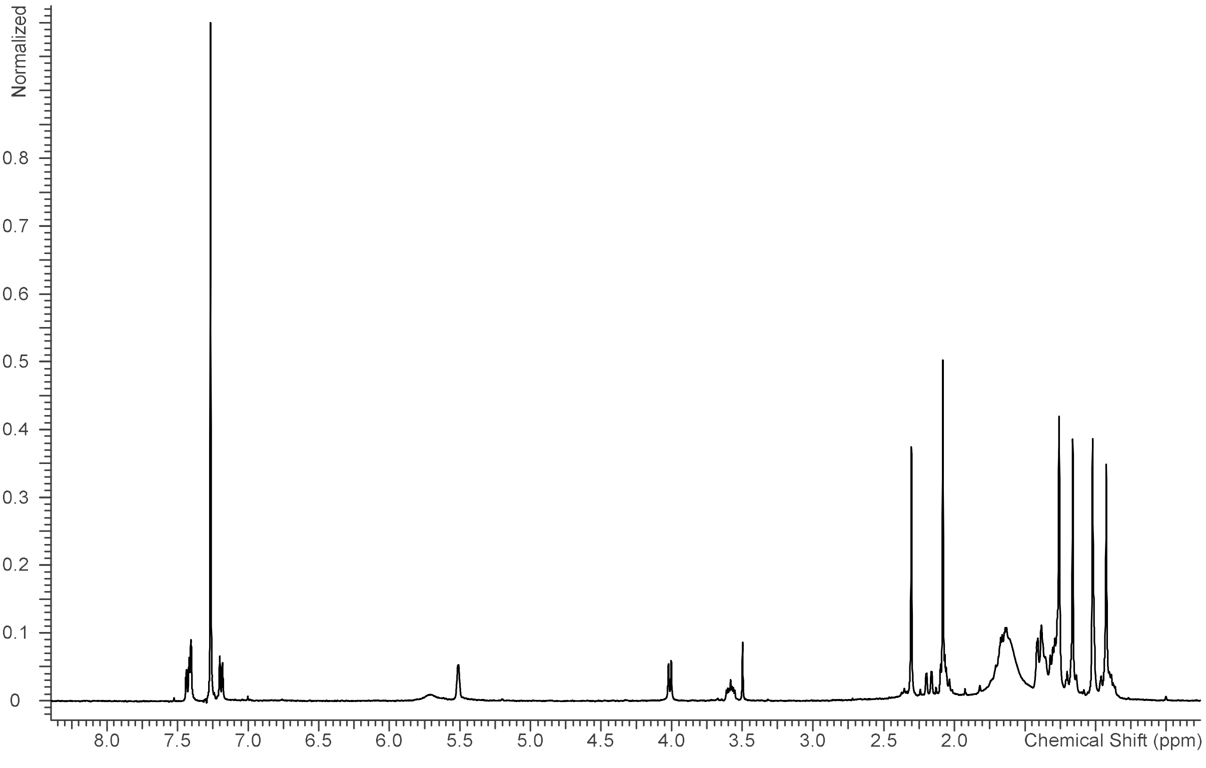

Figure S24 – ^{1}H NMR spectrum (400 MHz, $CDCl_3$) of **3**

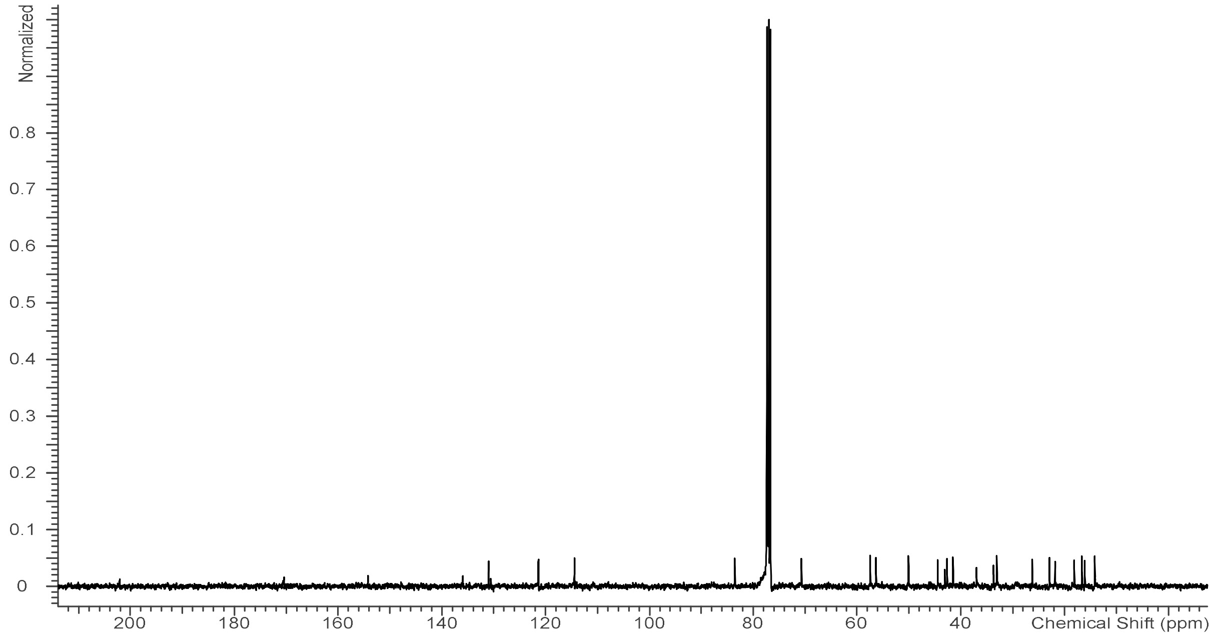

Figure S25 – ^{13}C NMR spectrum (100 MHz, $CDCl_3$) of **3**

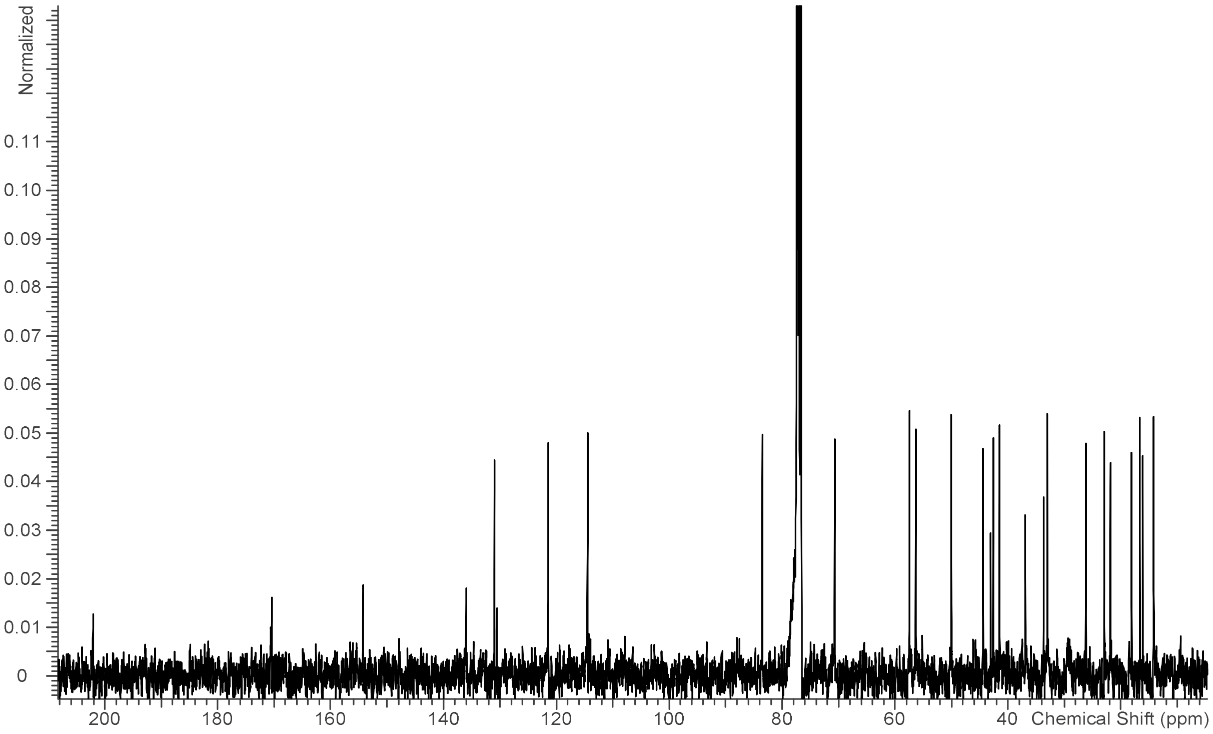

Figure S26 – ^{13}C NMR spectrum zoomed (100 MHz, $CDCl_3$) of **3**

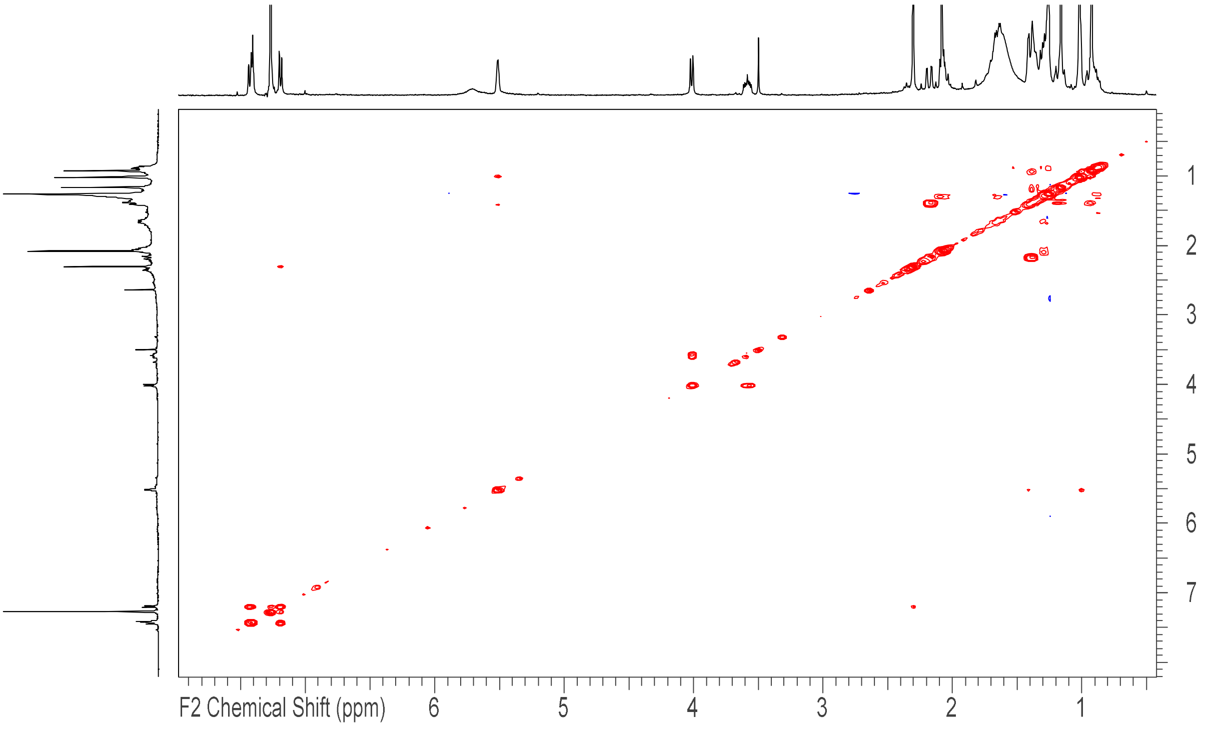

Figure S27 – COSY NMR spectrum (400 MHz, $CDCl_3$) of **3**

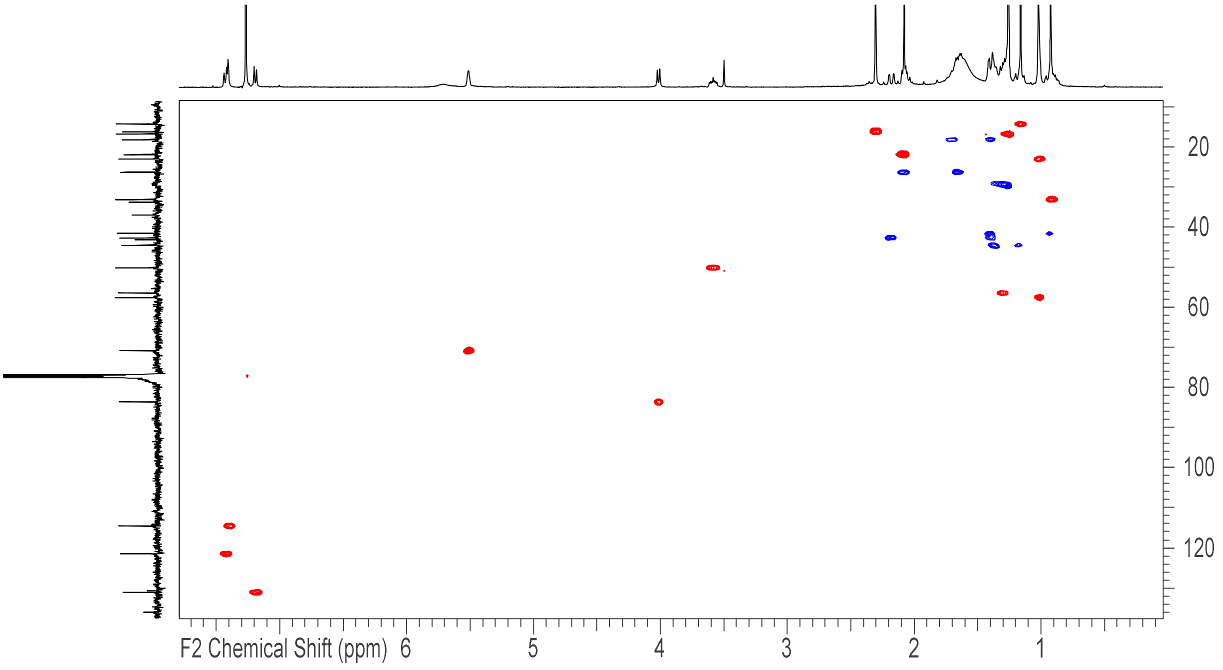

Figure S28 – HSQC NMR spectrum (400 MHz, $CDCl_3$) of **3**

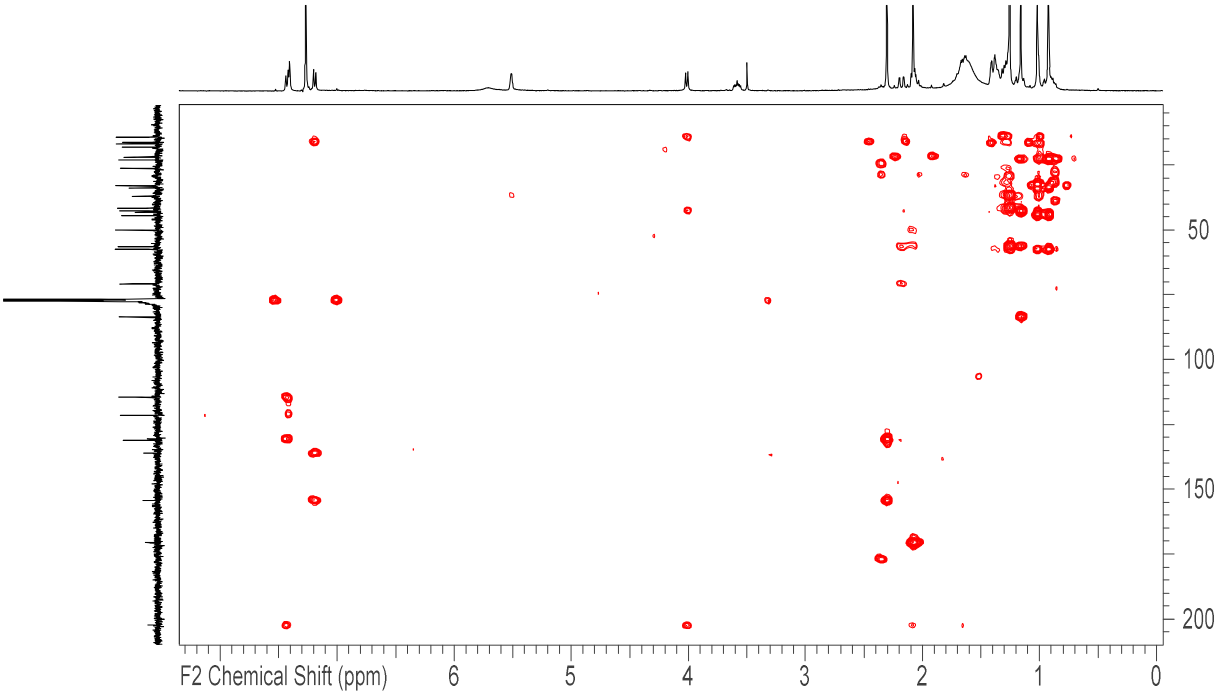

Figure S29 – HMBC NMR spectrum (400 MHz, $CDCl_3$) of **3**

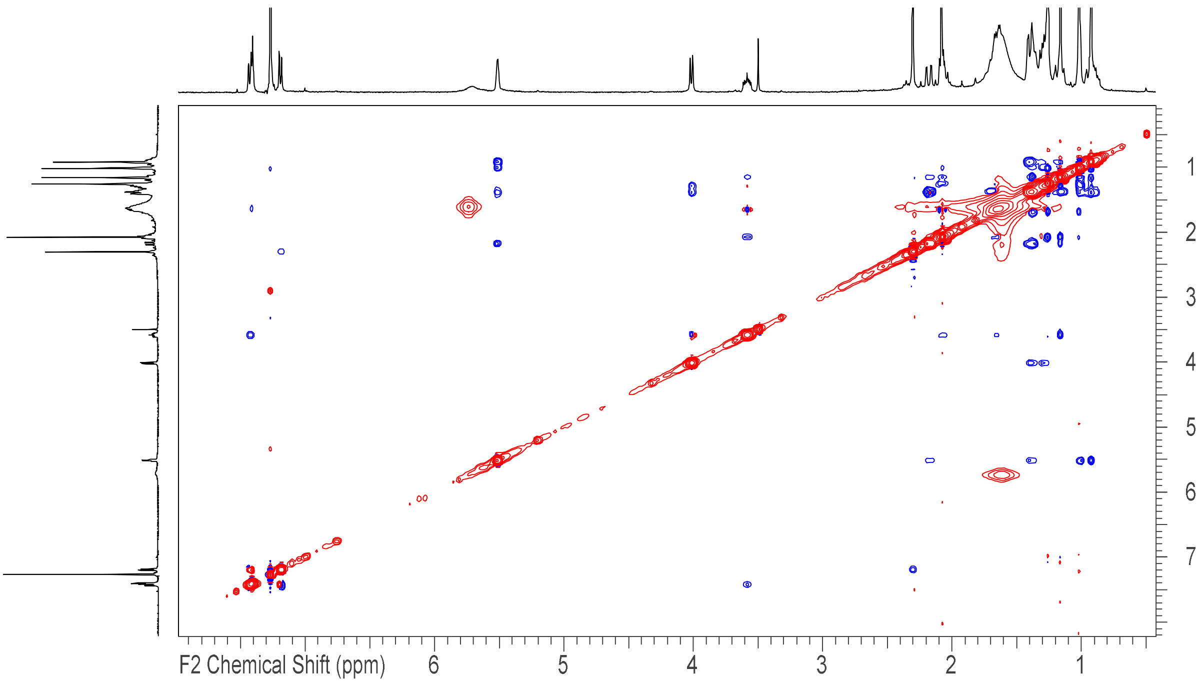

Figure S30 – NOESY NMR spectrum (400 MHz, $CDCl_3$) of **3**

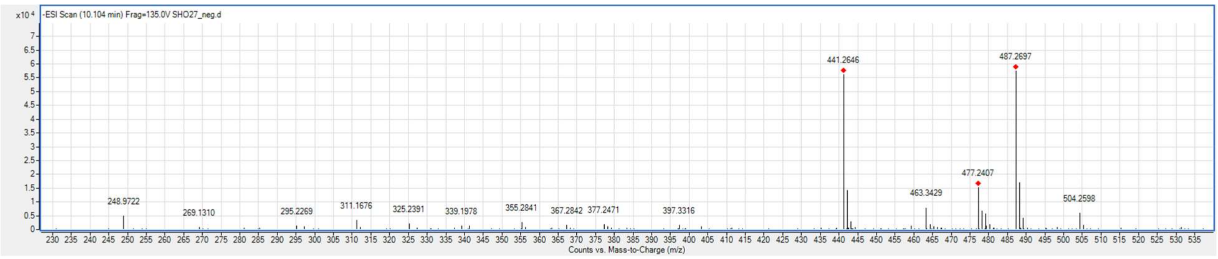

Figure S31 – HRESIMS analysis of **3**

Table S4 – NMR data for suberiphenol D (**4**) (400 (^{1}H) and 100 (^{13}C) MHz, ^{a}CDCl$_3$, ^{b}(CD$_3$)$_2$SO).

pos	$^a\delta_C$, type	$^b\delta_C$, type	$^a\delta_H$ (*J* in Hz)	$^b\delta_H$ (*J* in Hz)	gCOSY	gHMBC	^{a}Key NOESY	^{a}Key NOESY
**1**	155.3, C							
**2**	112.1, CH	111.1, CH	6.70, s	6.67, s	21	1, 4, 6, 21		
**3**	128.4, C							
**4**	148.4, C							
**5**	109.7, CH	109.7, CH	6.42, s	6.46, s		1, 3, 4, 7		
**6**	124.2, C							
**7**	83.3, C							
**8**	210.8, C							
**9a**	33.9, CH$_2$	34.1, CH$_2$	2.48, dd (19.0, 10.6)	2.25, dd (9.4, 9.4)	9b, 10	7, 8, 10, 15		
**9b**			2.33, dd (19.1, 9.0)	2.17, o/l	9a, 10	8		
**10**	45.9, CH	45.1, CH	1.88, dd (9.6, 9.6)	1.72, o/l	9a, 9b	9, 23, 24	14	
**11**	38.3, C							
**12a**	38.9, CH$_2$	38.5, CH$_2$	2.29, dd (15.1, 4.2)	2.12, ol	12b, 13			
**12b**			1.82, dd (15.0, 2.4)	1.69, o/l	12a, 13	10, 13, 14		
**13**	69.8, CH	68.9, CH	5.64, ddd (3.7, 2.1, 2.1)	5.49, br t (3.4)	12a, 12b, 14		14, 20	20
**14**	55.2, CH	54.5, CH	1.04, d (1.6)	0.99, o/l	13	15, 24, 25	10, 13	
**15**	37.4, C							
**16a**	41.8, CH$_2$	41.1, CH$_2$	1.37, o/l	1.35, o/l				
**16b**			0.76, ddd (12.2, 12.2, 2.8)	0.62, ddd (12.2, 12.2, 1.5)				
**17a**	18.1, CH$_2$	17.7, CH$_2$	1.62, m	1.54, m	17b			
**17b**			1.37, o/l	1.29, o/l	17a, 18b			
**18a**	43.6, CH$_2$	43.0, CH$_2$	1.36, o/l	1.28, o/l				
**18b**			1.17, dd (13.7, 2.5)	1.16, o/l	17b	25		
**19**	33.8, C							
**20**	33.1, CH$_3$	32.7, CH$_3$	0.98, s	0.91, s		19, 25	13	13
**21**	16.6, CH$_3$	16.6, CH$_3$	2.23, s	2.08, s	2	2, 3, 4		
**22**	97.4, CH	97.8, CH	4.19, s	4.02, s		1, 7, 8, 10, 11, 23	23	23, OH-7
**23**	18.9, CH$_3$	18.6, CH$_3$	1.00, s	0.94, s		10, 22	22, 27	22, OH-7
**24**	16.3, CH$_3$	16.0, CH$_3$	1.26, s	1.19, s		10, 14, 15	25	
**25**	23.2, CH$_3$	23.1, CH$_3$	1.00, s	0.94, s		18	24	
**26**	170.2, C							
**27**	21.8, CH$_3$	21.5, CH$_3$	2.06, s	2.02, s		26	23	23
**OH-4**				8.93, s				
**OH-7**				6.25, s				22, 23

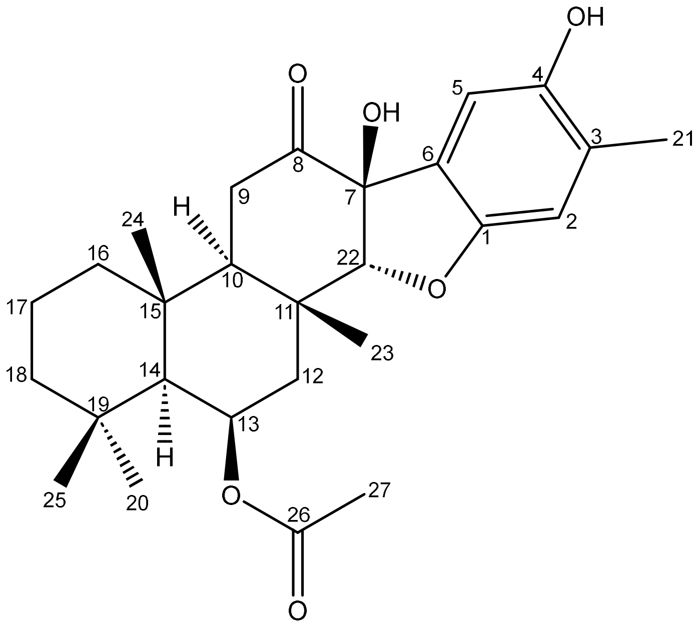

Figure S32 – Structure of **4**

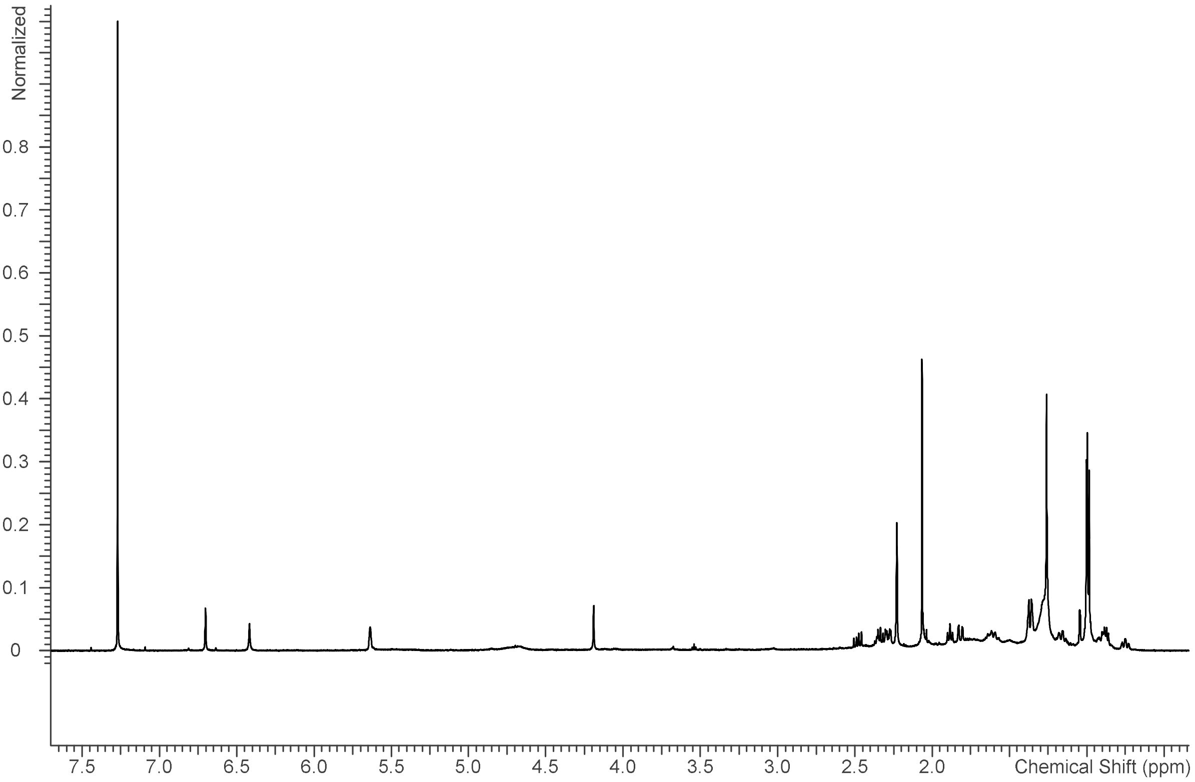

Figure S33 – ^{1}H NMR spectrum (400 MHz, $CDCl_3$) of **4**

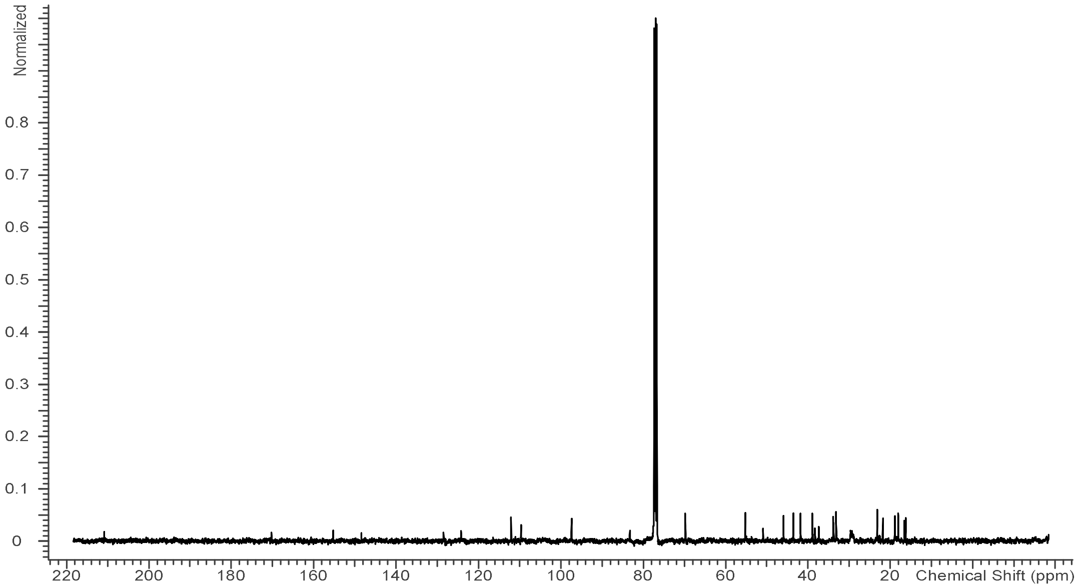

Figure S34 – ^{13}C NMR spectrum (100 MHz, $CDCl_3$) of **4** (δ_C 50.9, MeOH contaminant)

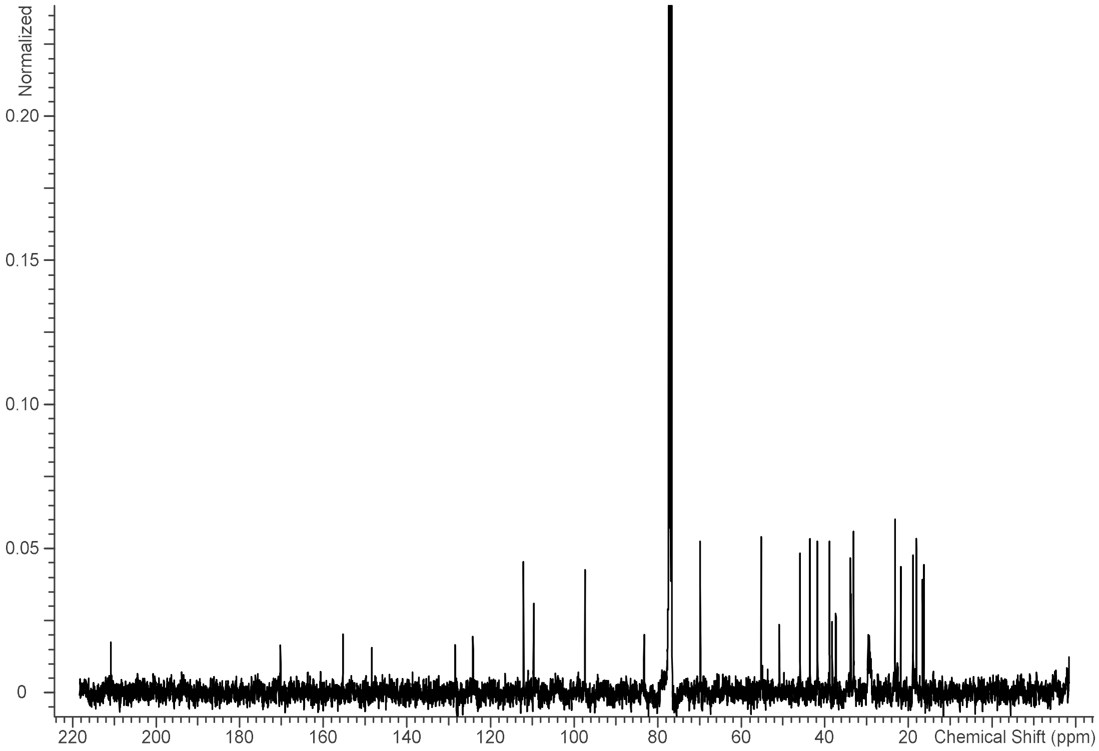

Figure S35 – ^{13}C NMR spectrum zoomed (100 MHz, $CDCl_3$) of **4** (δ_C 50.9, MeOH contaminant)

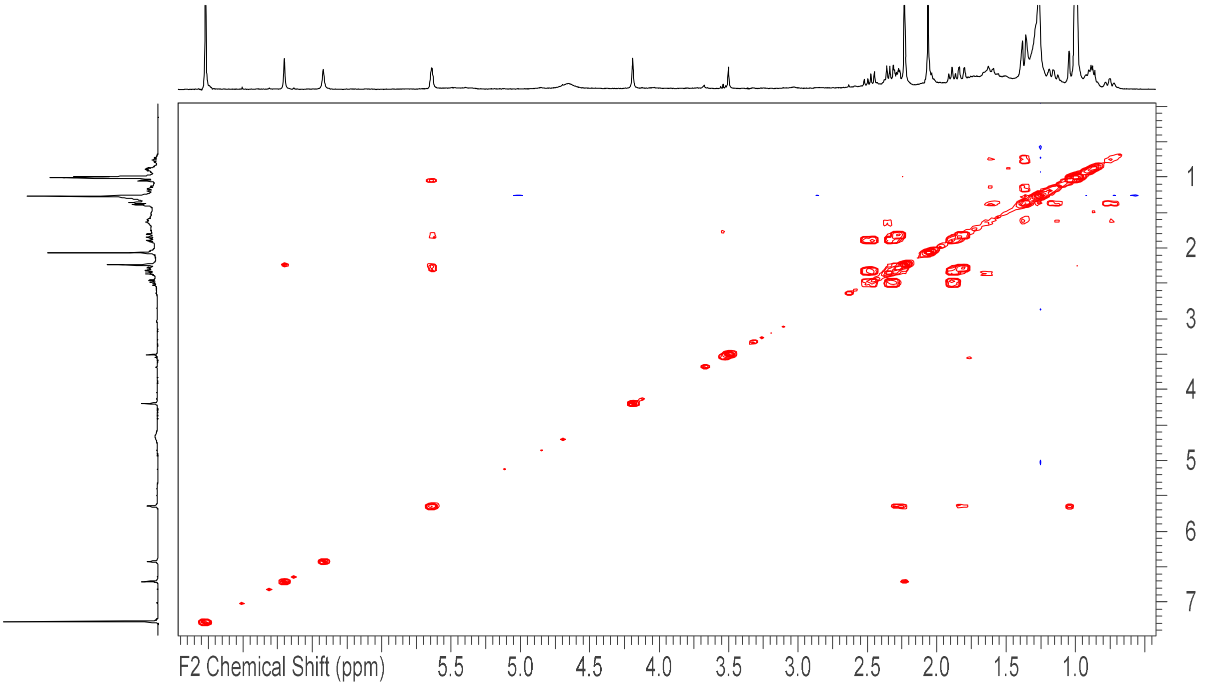

Figure S36 – COSY NMR spectrum (400 MHz, $CDCl_3$) of **4**

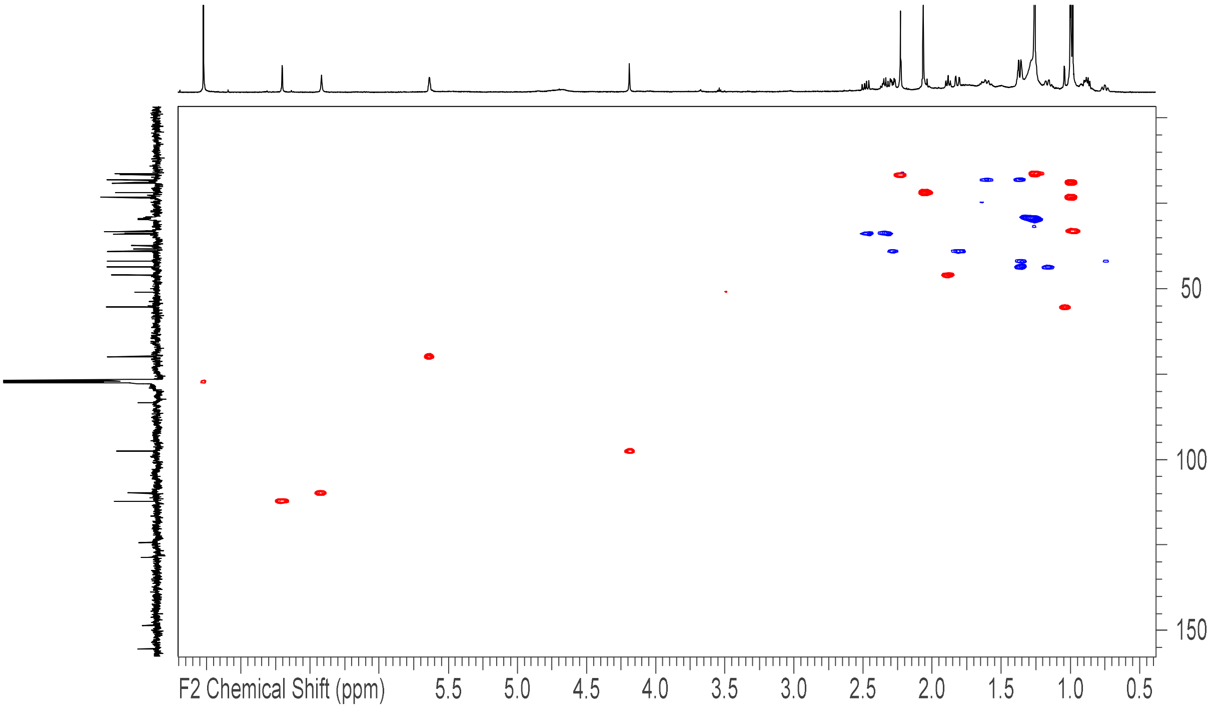

Figure S37 – HSQC NMR spectrum (400 MHz, $CDCl_3$) of **4**

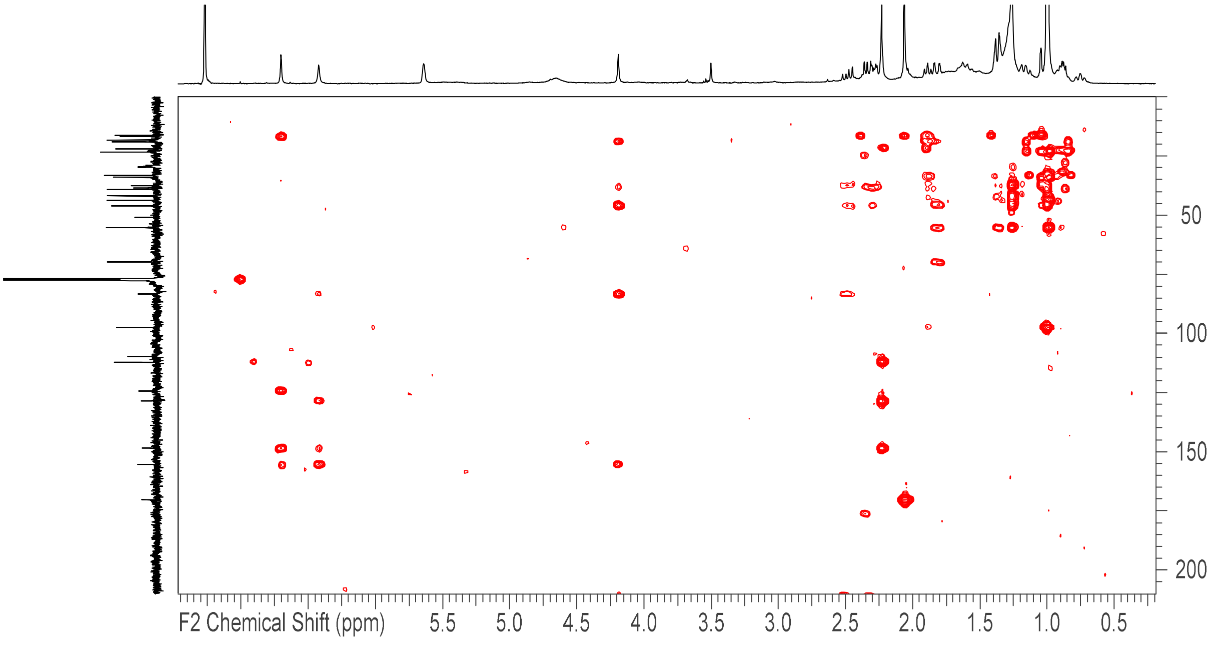

Figure S38 – HMBC NMR spectrum (400 MHz, $CDCl_3$) of **4**

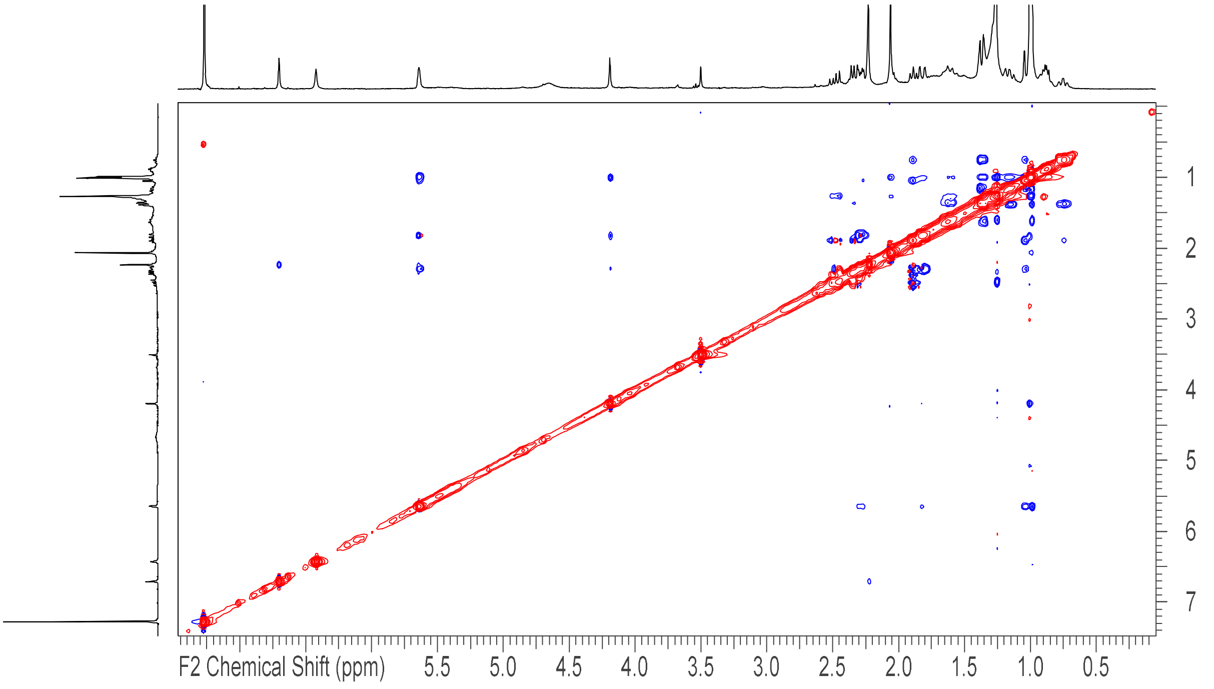

Figure S39 – NOESY NMR spectrum (400 MHz, $CDCl_3$) of **4**

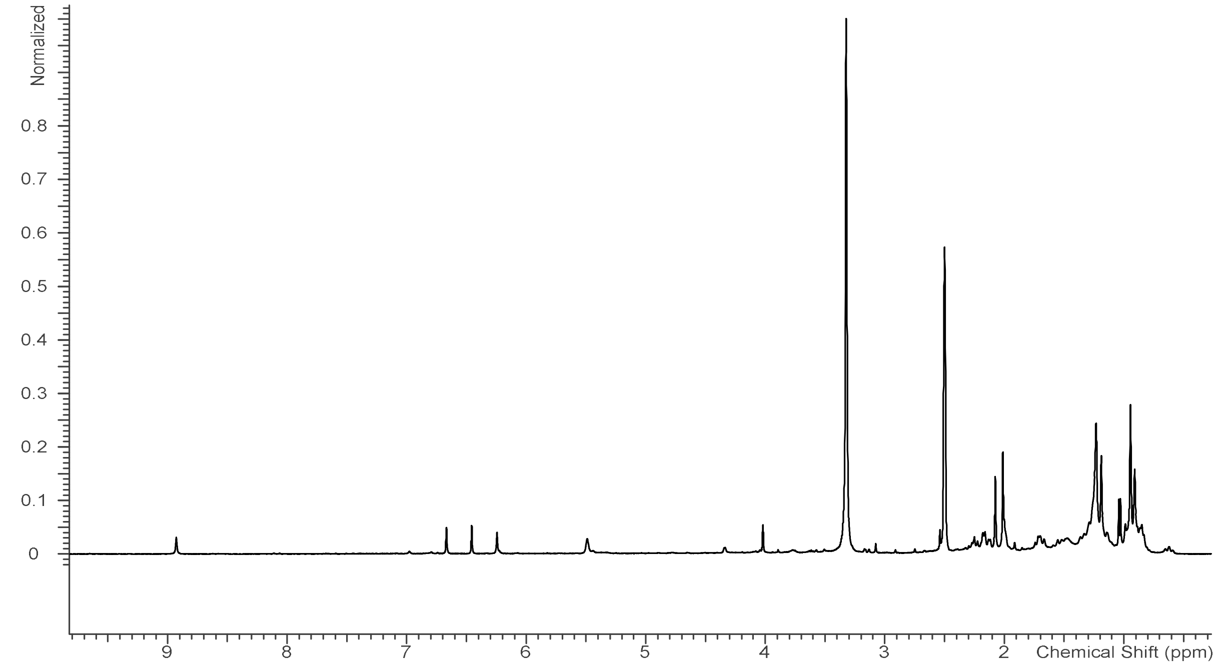

Figure S40 – ^{1}H NMR spectrum (400 MHz, $(CD_3)_2SO$) of **4**

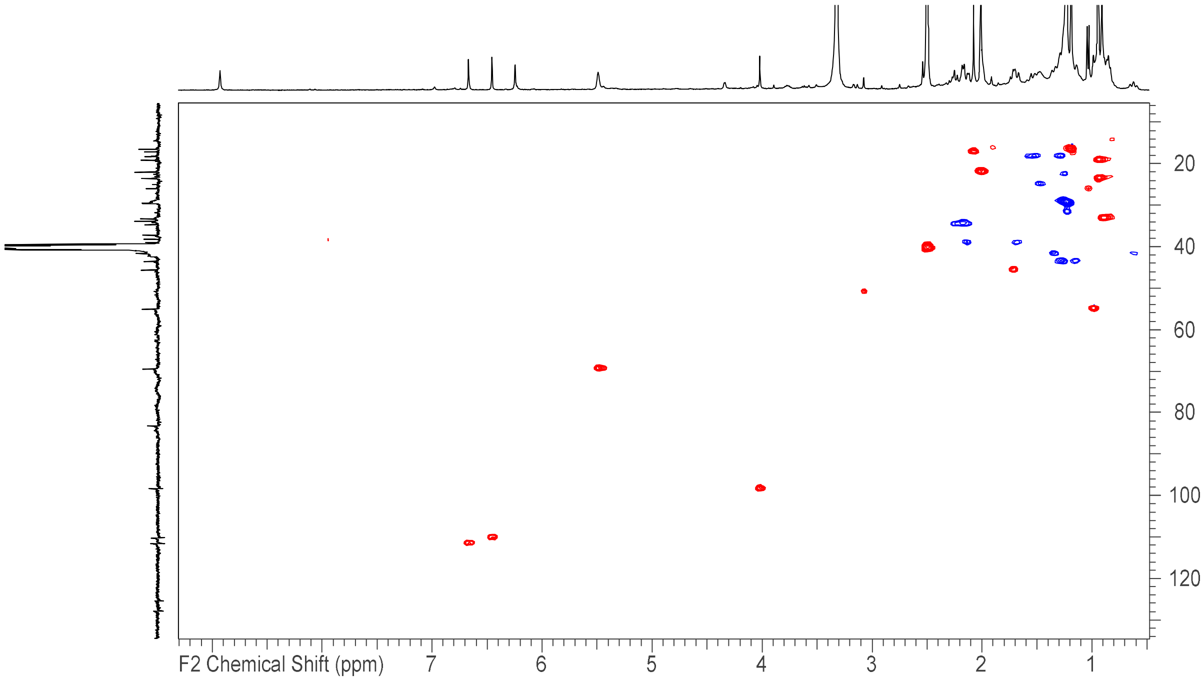

Figure S41 – HSQC NMR spectrum (400 MHz, $(CD_3)_2SO$) of **4**

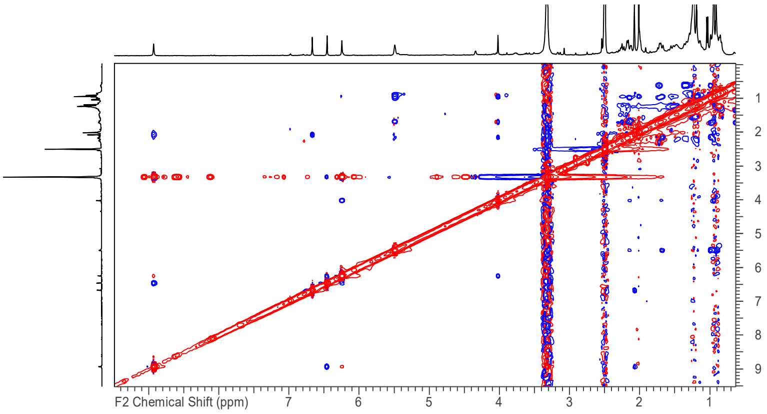

Figure S42 – NOESY NMR spectrum (400 MHz, $(CD_3)_2SO$) of **4**

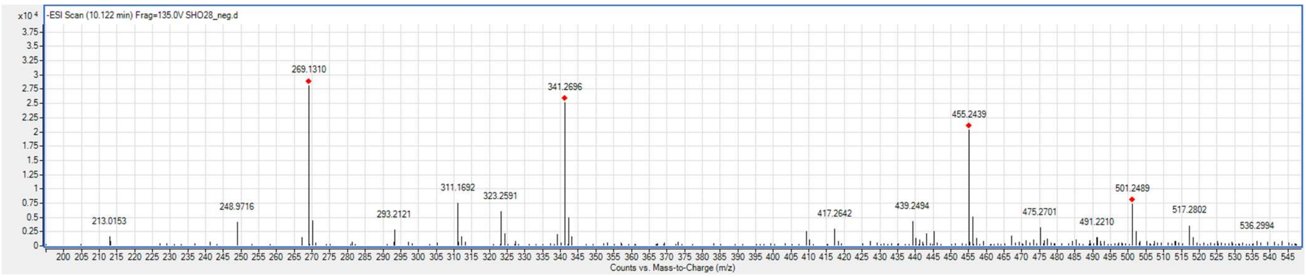

Figure S43 – HRESIMS analysis of **4**

Table S5 – NMR data for suberone (**5**) (400 (^{1}H) and 100 (^{13}C) MHz, $CDCl_3$).

pos	δ_C, type	δ_H (*J* in Hz)	gCOSY	gHMBC	Key NOESY
**1a**	59.6, CH_2	2.10, o/l	1b, 16	6, 7, 16	
**1b**		2.03, o/l	1a	2, 5, 6	
**2**	210.9, C				
**3a**	41.6, CH_2	2.44, dddd (14.5, 4.4, 2.2, 2.2)	4b		
**3b**		2.29, dddd (13.9, 13.9, 7.1, <0.5)	4a	2	
**4a**	18.5, CH_2	1.74, o/l			
**4b**		1.50 o/l			
**5**	56.6, CH	1.50 o/l			9
**6**	37.9, C				
**7a**	45.7, CH_2	1.91, dd (2.8, 14.9)	7b, 8	6, 8, 9	
**7b**		1.51 o/l	7a, 8		
**8**	70.0, CH	5.52, ddd (2.9, 2.5, 2.5)	7a, 7b, 9	6, 7	9, 15
**9**	56.5, CH	1.15, d (1.9)	8	6, 14, 17, 18	5, 15
**10**	37.7, C				
**11a**	42.3, CH_2	1.82, o/l	11b		
**11b**		1.00, o/l	11a		
**12**	22.0, CH_2	1.79, o/l		9, 10, 11	
**13a**	44.0, CH_2	1.41, ddd (12.9, 3.8, 3.7)		11	
**13b**		1.22, o/l			
**14**	34.0, C				
**15**	33.2, CH_3	0.97, s		9, 13, 14, 18	8, 9
**16**	21.2, CH_3	1.06, s	1a	1, 5, 6, 7	20
**17**	17.0, CH_3	1.23, s		5, 10, 11	18
**18**	23.1, CH_3	1.02, s		9, 13, 14, 15	17
**19**	170.3, C				
**20**	21.7, CH_3	2.05, s		19	16

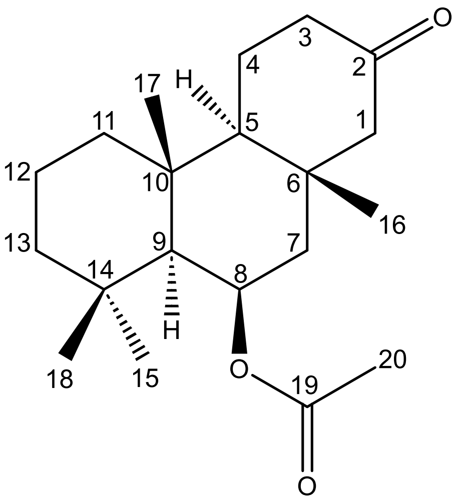

Figure S44 – Structure of **5**

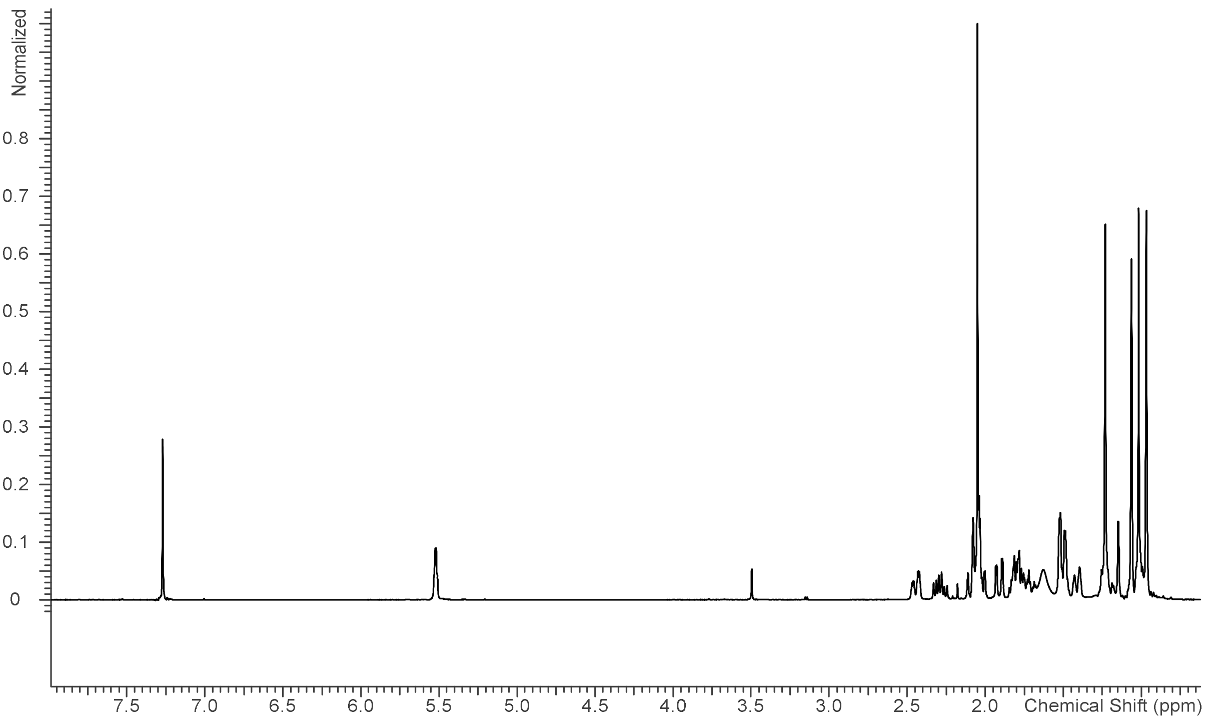

Figure S45 – ^{1}H NMR spectrum (400 MHz, $CDCl_3$) of **5**

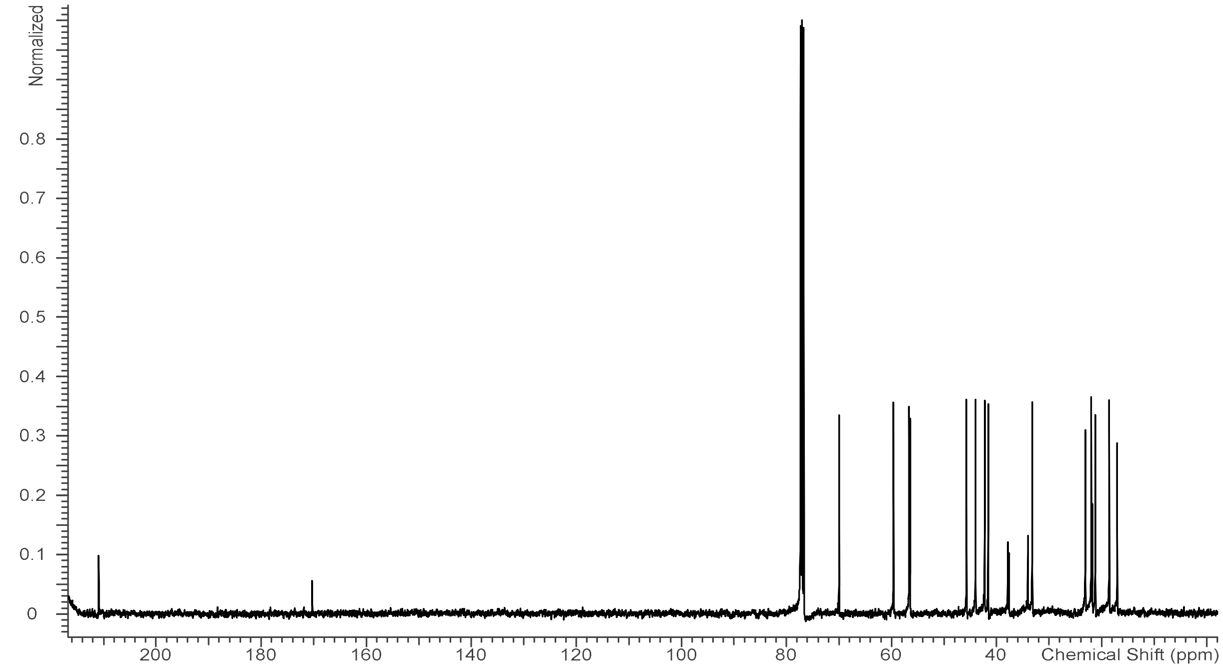

Figure S46 – ^{13}C NMR spectrum (100 MHz, $CDCl_3$) of **5**

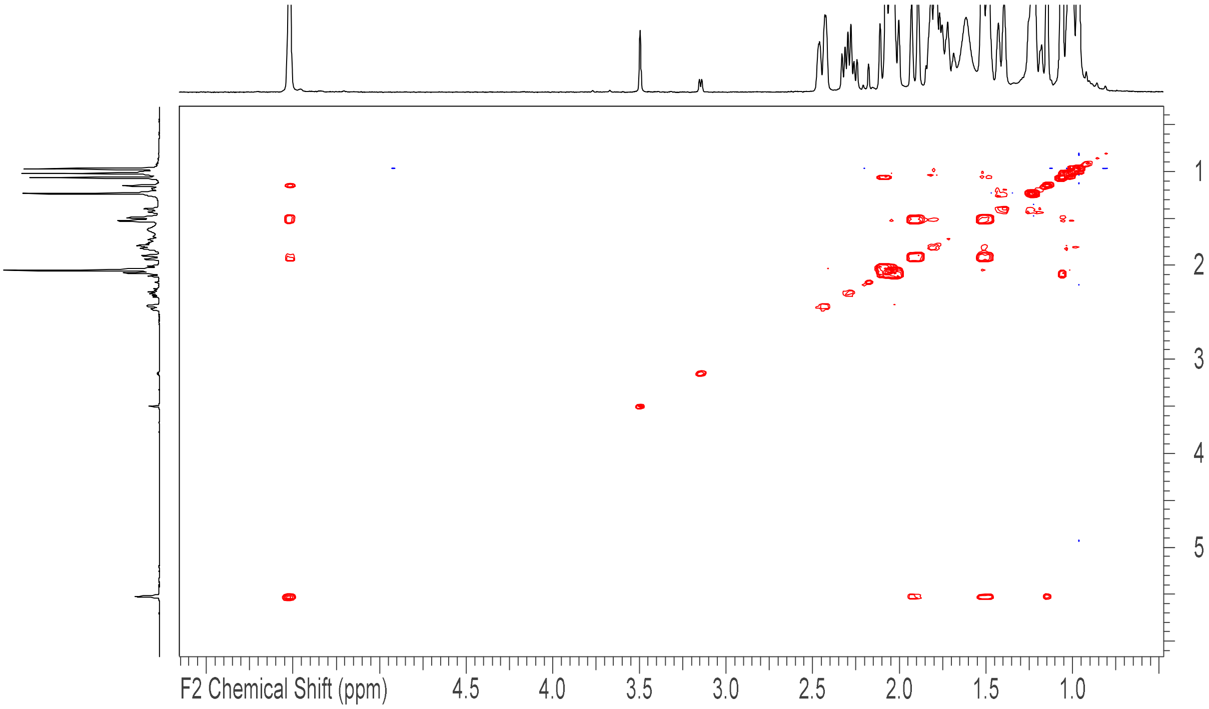

Figure S47 – COSY NMR spectrum (400 MHz, $CDCl_3$) of **5**

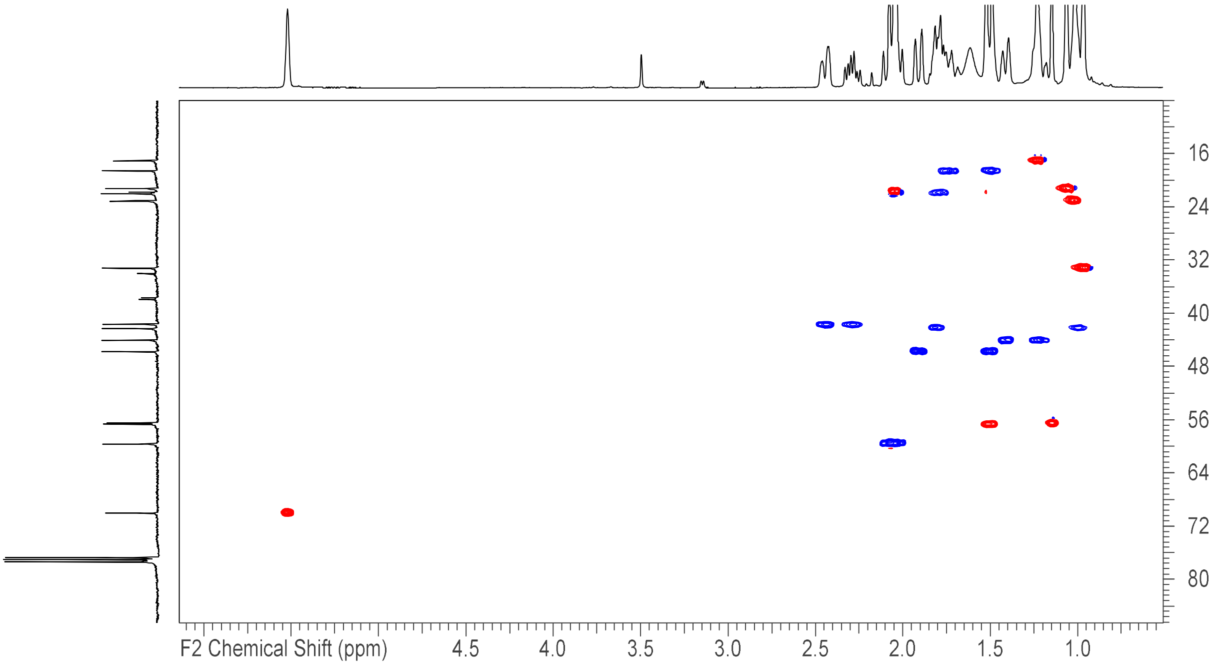

Figure S48 – HSQC NMR spectrum (400 MHz, $CDCl_3$) of **5**

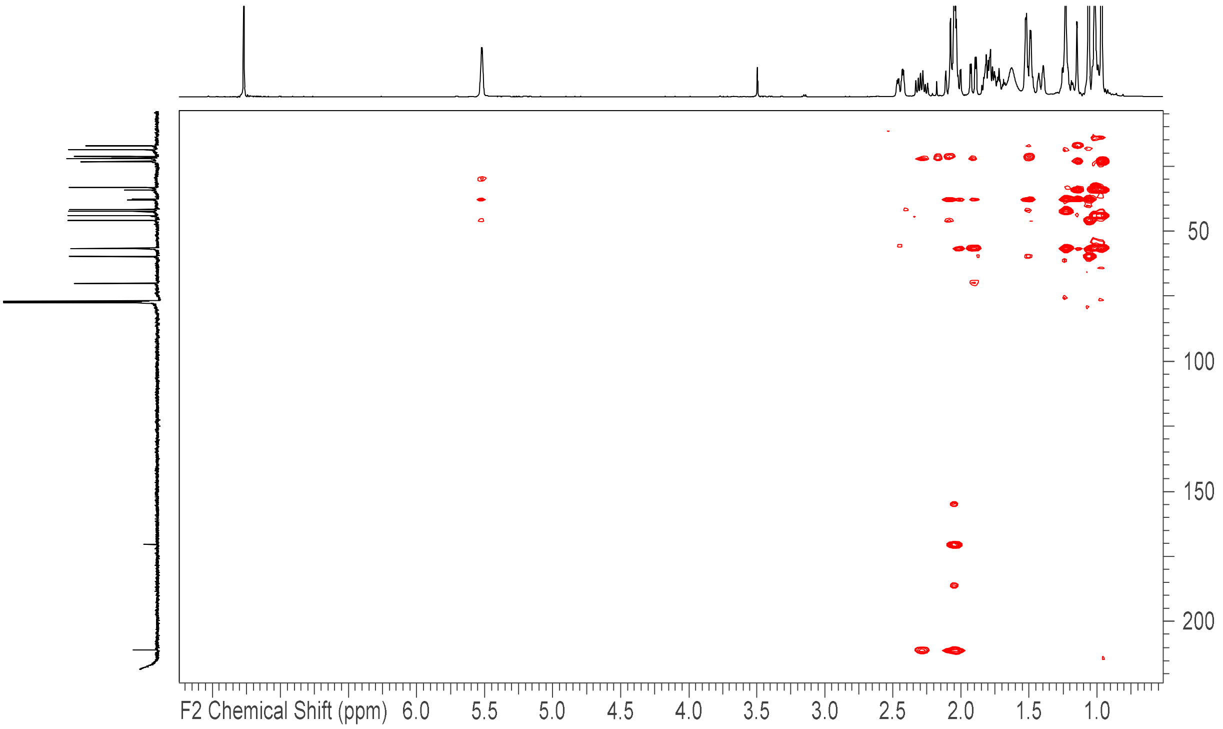

Figure S49 – HMBC NMR spectrum (400 MHz, $CDCl_3$) of **5**

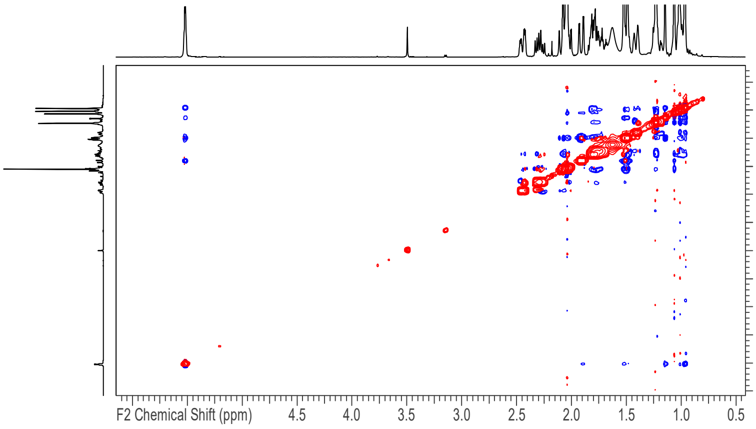

Figure S50 – NOESY NMR spectrum (400 MHz, $CDCl_3$) of **5**

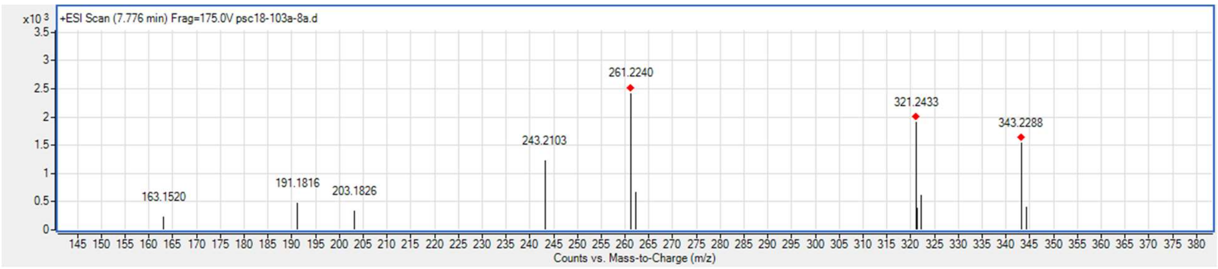

Figure S51 – HRESIMS analysis of **5**

Table S6 – Crystal Data and Structure Refinement for Suberone (**5**)

Identification code	Suberone
Empirical formula	$C_{20}H_{32}O_3$
Formula weight	320.45
Temperature/K	100.00
Crystal system	orthorhombic
Space group	$P2_12_12_1$
a/Å	6.1782(2)
b/Å	15.1168(4)
c/Å	19.2774(5)
α/°	90
β/°	90
γ/°	90
Volume/Å^3	1800.41(9)
Z	4
ρ_{calc}g/cm^3	1.182
μ/mm^{-1}	0.607
F(000)	704.0
Crystal size/mm^3	0.22 × 0.05 × 0.03
Radiation	CuKα (λ = 1.54178)
2Θ range for data collection/°	7.432 to 158.05
Index ranges	$-7 \le h \le 7$, $-18 \le k \le 19$, $-24 \le l \le 24$
Reflections collected	26268
Independent reflections	3834 [R_{int} = 0.0472, R_{sigma} = 0.0266]
Data/restraints/parameters	3834/0/213
Goodness-of-fit on F^2	1.064
Final R indexes [I>=2σ (I)]	R_1 = 0.0300, wR_2 = 0.0795
Final R indexes [all data]	R_1 = 0.0316, wR_2 = 0.0807
Largest diff. peak/hole / e Å^{-3}	0.23/-0.16

Flack parameter	0.05(6)

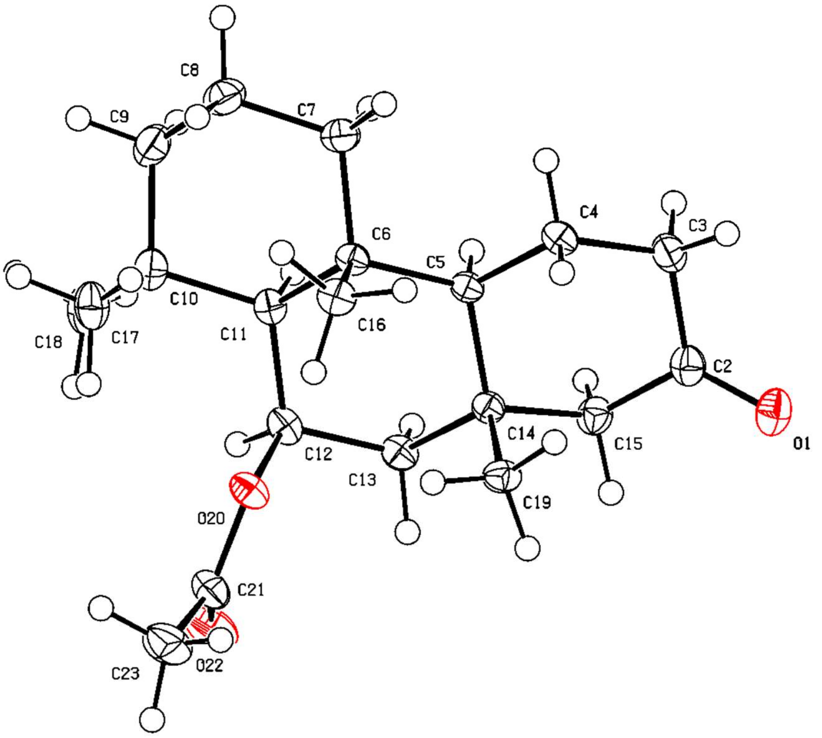

Figure S52 – Ellipsoid plot of **5**. Anisotropic displacement parameters were drawn at 50% probability level.

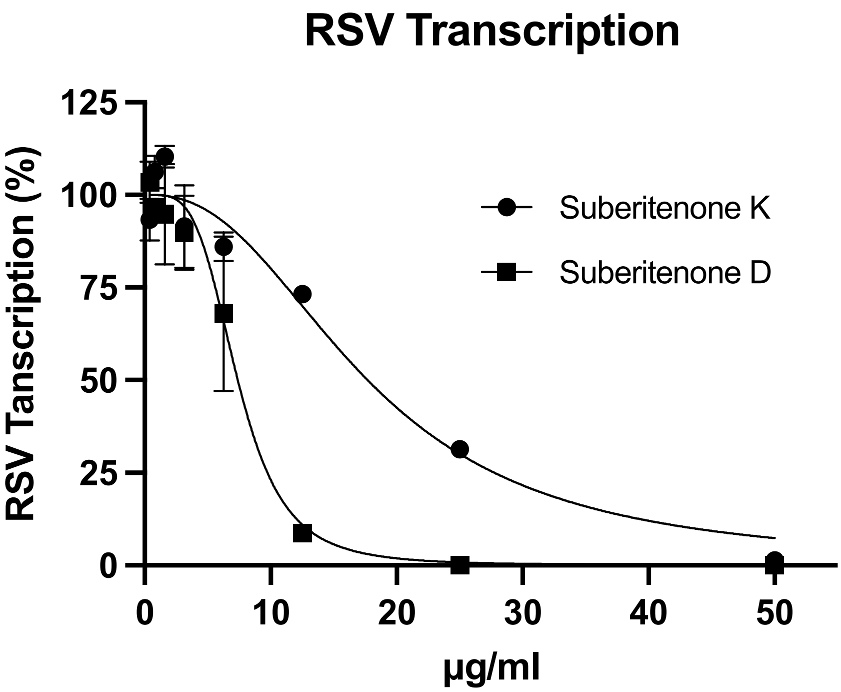

Figure S53 – RSV Transcription vs [treatment] curve for suberitenone K (**1**) and suberitenone D (**9**).

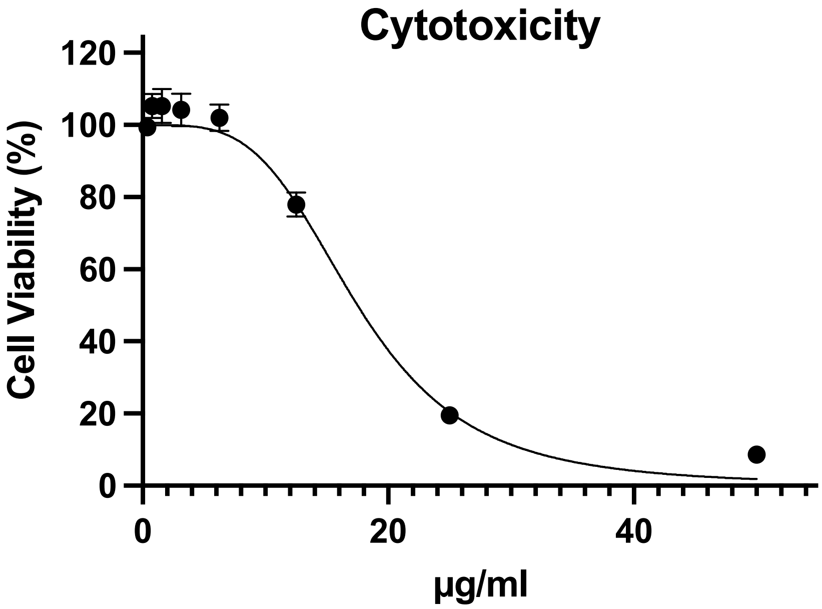

Figure S54 – A549 Cell viability vs [treatment] curve for suberitenone D (**9**).
